# Cellulosic Functional Bioplastic with Tunable Strength and Toughness Through Heat‐Treatment of Dynamic Covalent Networks

**DOI:** 10.1002/advs.202508075

**Published:** 2025-07-21

**Authors:** Xiangyu Tang, Linlin Zhao, Yunfeng Guo, Ying Wang, Zhenke Wei, Xinyan Fan, Zefang Xiao, Haigang Wang, Yanjun Xie, Yonggui Wang

**Affiliations:** ^1^ Key Laboratory of Bio‐based Material Science and Technology (Ministry of Education) College of Material Science and Engineering Northeast Forestry University Hexing 26 Road Harbin 150040 P. R. China

**Keywords:** cellulose, diels‐alder reaction, heat‐treatment, lignin, tunable mechanical

## Abstract

The growing environmental crisis caused by petroleum‐based polymers has intensified the development of sustainable alternatives, with many biomass‐derived polymers demonstrating potential for degradability, renewability, and low carbon footprints, though these properties can vary depending on structure and processing. However, traditional bio‐based systems often lack tunability in mechanical properties, making it challenging to achieve both high strength and ductility. Herein, we report a high‐performance, recyclable bio‐based film (CAF‐L) constructed via Diels‐Alder dynamic covalent chemistry between furfuryl‐functionalized cellulose acetate and maleimide‐modified lignin. Thermally responsive dynamic Diels‐Alder bonds, activated through heat‐treatment, enable programmable network crosslinking that allows a smooth transition between strength‐ and ductility‐dominated regimes, while maintaining high mechanical performance (tensile strength up to 52.3 MPa and elongation at break up to 545%). Structural characterization and molecular simulations reveal that Diels‐Alder bond dynamics drive thermally induced structural reorganization of the polymer network, imparting rare adaptivity to biomass‐based systems. In addition, CAF‐L films exhibit outstanding UV shielding, oxygen barrier properties, and dual‐mode recyclability through solvent dissolution and hot pressing. This work provides a scalable platform for constructing mechanically tunable, structurally reconfigurable, and environmentally resilient cellulosic bioplastic for sustainable packaging and circular material systems.

## Graphical abstract

A fully bio‐based dynamic covalent network is constructed via Diels‐Alder chemistry between furan‐modified cellulose derivatives and bismaleimide‐functionalized lignin. This system enables tunable strength‐ductility balance through heat‐treatment and exhibits excellent recyclability. The strategy provides a sustainable route for high‐performance bioplastics from renewable biomass resources.

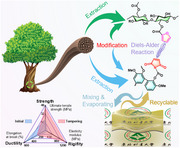

## 1 Introduction

The widespread use of petroleum‐based plastics has led to severe environmental issues, including long‐term microplastic accumulation, carbon emissions, and limited recyclability.^[^ ^1^, ^2^ ^]^ In response, there is a growing demand for sustainable alternatives that are both renewable and environmentally benign. Bio‐based plastics have emerged as a promising class of materials in this context. Derived from natural resources such as cellulose, lignin, starch, and polylactic acid, they can offer biodegradability and a lower carbon footprint depending on feedstock and processing methods.^[^ ^3^, ^4^, ^5^ ^]^ In particular, cellulose and lignin, the two primary components of lignocellulosic biomass, are especially attractive due to their abundance, chemical functionality, and structural complementarity.^[^ ^6^, ^7^, ^8^ ^]^

Cellulose, the most abundant biopolymer on Earth, offers excellent biodegradability, chemical modifiability, and film‐forming ability, making it an ideal matrix for bio‐based plastics. As a linear polysaccharide, it provides flexibility and processability to polymer systems.^[^ ^9^ ^]^ Among its many derivatives, cellulose acetate has been widely applied in sustainable film materials due to its improved solubility and thermoplastic processing properties.^[^ ^10^, ^11^, ^12^ ^]^ Lignin, the second most abundant biopolymer, possesses a unique aromatic structure that imparts UV shielding, thermal resistance, and mechanical rigidity.^[^ ^13^, ^14^ ^]^ However, its structural heterogeneity often limits its compatibility with cellulose‐based matrices, necessitating the use of chemical modification strategies to construct integrated networks.^[^ ^15^, ^16^ ^]^

In recent years, dynamic covalent chemistry, particularly the Diels‐Alder reaction between furan and maleimide groups, has emerged as a powerful strategy for constructing structurally reconfigurable and functionally tunable polymer networks.^[^ ^17^, ^18^, ^19^ ^]^ The Diels‐Alder reaction enables thermally reversible crosslinking under mild conditions, endowing materials with adjustable mechanical performance, self‐healing capabilities, and recyclability.^[^ ^20^, ^21^, ^22^, ^23^ ^]^ While Diels‐Alder chemistry has been extensively explored in synthetic systems, its integration into fully bio‐based platforms remains an evolving area. Nevertheless, several recent efforts have demonstrated successful adaptation of Diels‐Alder chemistry to lignin‐containing systems, constructing nanophase‐separated networks with enhanced mechanical properties and recyclability by leveraging lignin's aromatic rigidity and thermal resistance.^[^ ^24^ ^]^ The adaptability of Diels‐Alder networks has also been demonstrated in systems where healing occurs even in vitrified states, indicating that mobility‐controlled Diels‐Alder reactions can operate across a broad temperature range.^[^ ^25^ ^]^ Furthermore, Diels‐Alder‐based reversible adhesives, such as bioaromatic “superglues” developed from lignin derivatives, showcase the versatility of this chemistry for robust yet sustainable materials with closed‐loop circularity.^[^ ^26^ ^]^ Moderate heat‐treatment below the degradation threshold has been shown to effectively regulate the reversibility of Diels‐Alder linkages, enabling dynamic rearrangement of the network architecture and tunable crosslinking density.^[^ ^27^ ^]^ Such temperature‐controlled strategies, when integrated with dynamic covalent chemistry, offer a promising pathway toward recyclable and structurally adaptive bio‐based polymer networks.

In this work, we present a fully biomass‐derived, dynamically cross‐linked film synthesized from furfuryl‐modified cellulose acetate (CAF) and maleimide‐functionalized lignin (LMA). By adjusting the heat‐treatment conditions, the crosslinking density and mechanical properties can be precisely tuned. Here, heat‐treatment refers to a thermodynamically governed process that selectively activates the forward Diels‐Alder reaction between furan and maleimide groups to modulate the crosslinking density within the polymer network. The films exhibit tunable mechanical properties, with tensile strength increasing to 52.3 MPa and elongation at break decreasing from 545% to 50% as crosslinking increases. This tunable balance between strength and elongation highlights the programmability of the network structure. Structural analyses, including SAXS and molecular simulations, reveal thermally responsive network rearrangement driven by dynamic Diels‐Alder linkages, highlighting a unique strategy for programming mechanical behavior in fully bio‐based systems.

## 2 Results and Discussion

**Figure 1:**
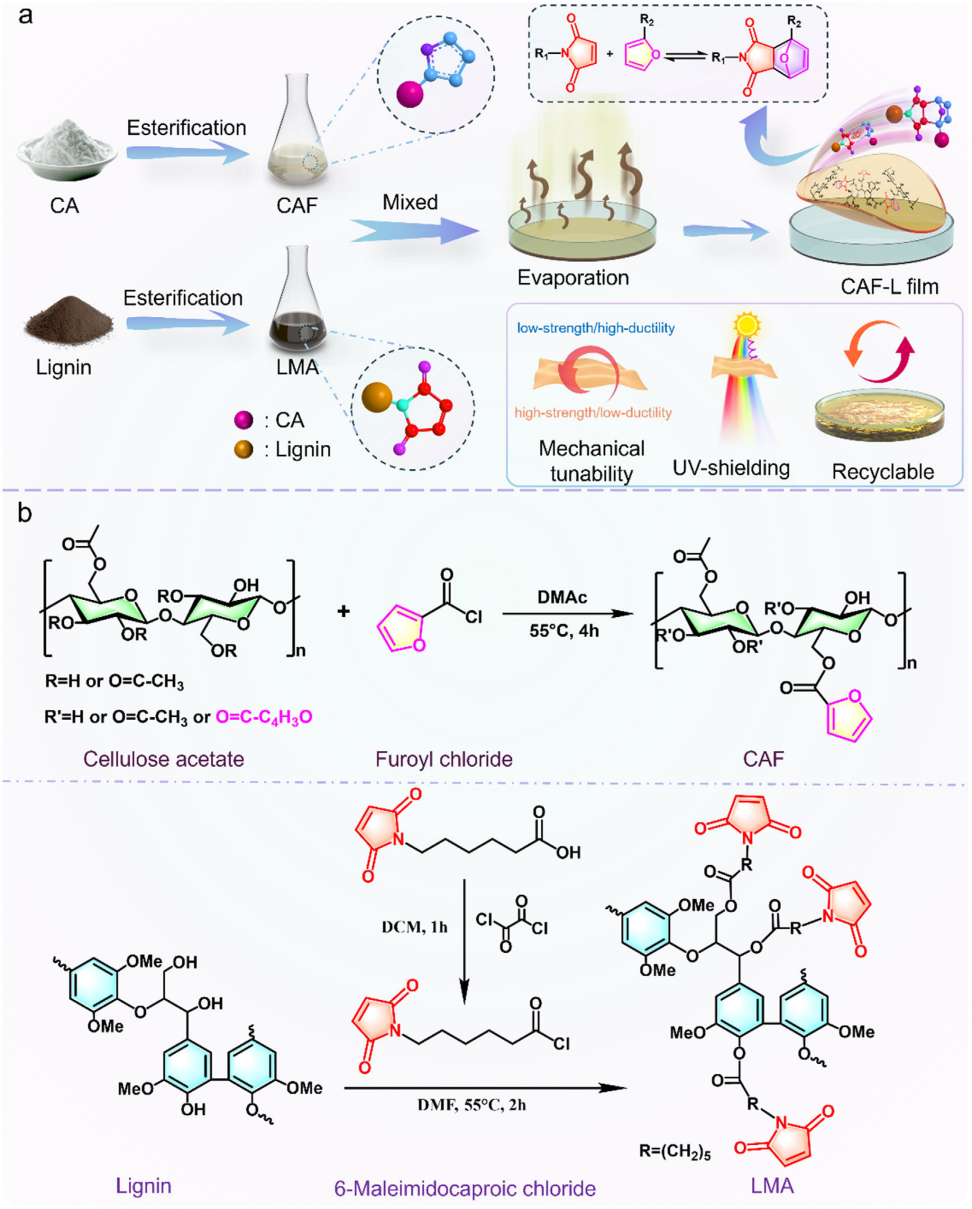
a) Schematic diagram of the preparation process of CAF‐L films. b) Synthetic reaction equations for CAF and LMA.

To fabricate a dynamically cross‐linked, bio‐based polymeric system with tailored mechanical properties and reconfigurable structure, we developed a Diels‐Alder strategy based on functionalized cellulose acetate and lignin. As schematically illustrated in **Figure** **1**a, the fabrication process begins with the chemical modification of cellulose acetate and lignin via esterification with furfuryl chloride and 6‐maleimidocaproyl chloride, respectively. These reactions introduce thermoresponsive furan and maleimide groups into the polymer backbones, enabling the formation of reversible covalent linkages. The reaction mechanisms and structural transformations involved in the functionalization steps are detailed in Figure 1b. Upon homogeneous blending of the two precursors in tetrahydrofuran and subsequent solvent evaporation, the furan and maleimide moieties undergo thermally induced Diels‐Alder cycloaddition to form a dynamic covalent network. This cross‐linked architecture not only stabilizes the CAF‐L film but also allows for tunable cross‐linking density through post‐treatment, thereby enabling precise modulation of mechanical properties. The reversible nature of the Diels‐Alder bonds further imparts recyclability and thermal reprocess ability to the material, laying the foundation for a scalable and sustainable platform for next‐generation biomass‐derived functional films.

### 2.1 Preparation of CAF and LMA

**Figure 2:**
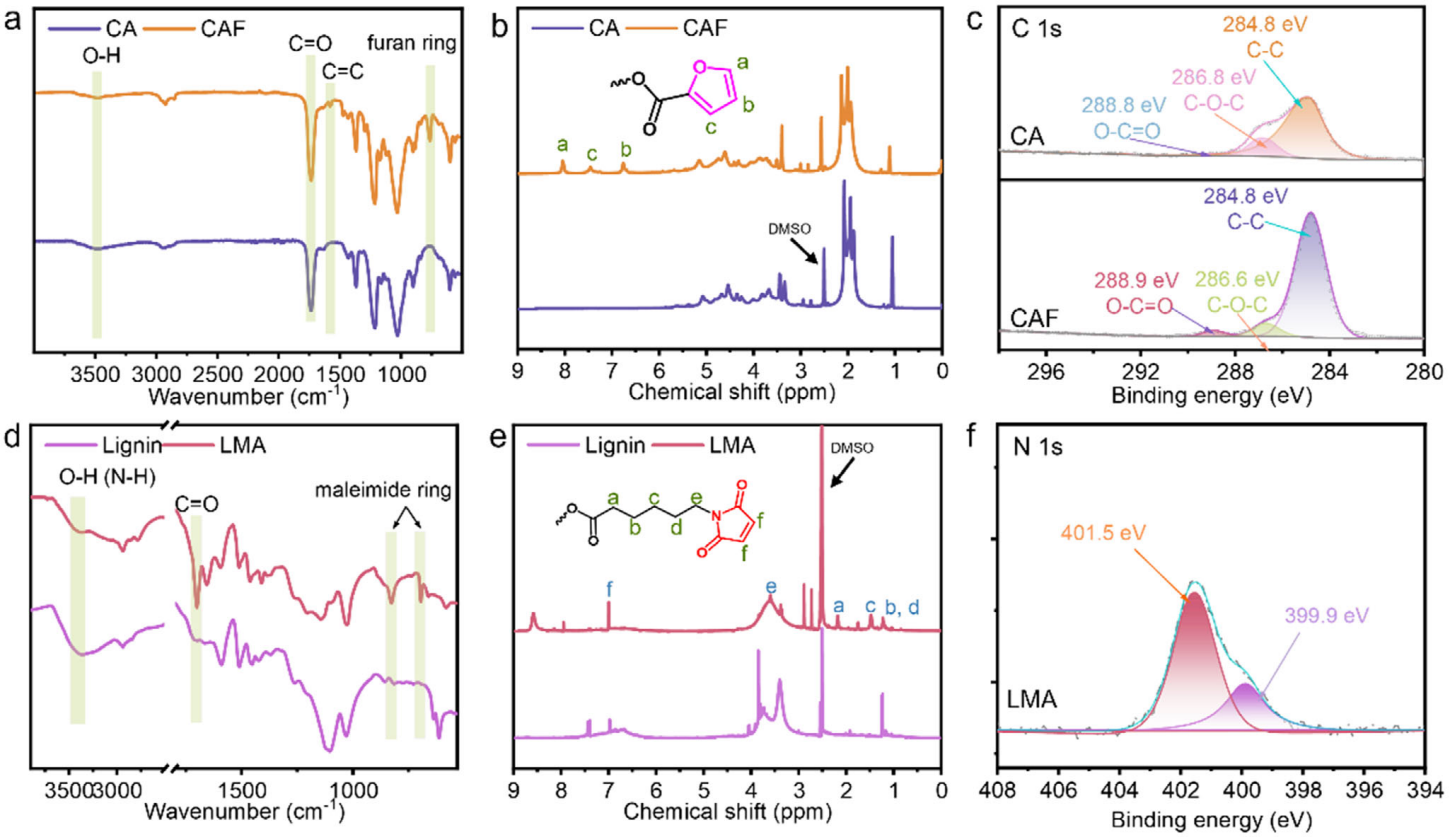
Chemical structures of CAF and LMA. a,d) FTIR spectra, b,e) liquid‐state ^1^H NMR spectra, c) high‐resolution C 1s XPS spectra of CA and CAF, f) high‐resolution N 1s XPS spectra of LMA.

This study investigates the modification of cellulose acetate and lignin, as outlined by the reaction equation presented in Figure 1b. Cellulose acetate is modified under homogeneous conditions using furfuryl chloride, where hydroxyl groups undergo nucleophilic substitution in the presence of pyridine as a catalyst. The successful modification is confirmed by the infrared spectrum shown in **Figure** **2**a. This spectrum shows enhanced absorption peaks for C═C bonds and furan rings, as well as a marked increase in the C═O bond peaks compared to their unmodified state.^[^ ^22^ ^]^ The ^1^H NMR spectrum reveals a distinct signal between 6.5 and 8 ppm, corresponding to the hydrogen atoms on the furan ring, confirming the incorporation of the furan group in CAF (Figure 2b). Additionally, the ^13^C NMR spectrum reveals a significant increase in the signal intensity of the carbonyl group, along with the appearance of signals from the carbon atoms on the furan ring (Figure S1, Supporting Information).^[^ ^28^ ^]^ The XPS spectrum, depicted in Figure S2 (Supporting Information), reveals a notable increase in the intensity of the C1s derived peaks from CAF. Fractionating the C1s diffraction peaks before and after modification highlights a substantial intensification at the O─C═O peak at 288.8 eV, validating the successful synthesis of CAF as illustrated in Figure 2c.

Due to the low reactivity between the phenolic hydroxyl groups of lignin and the carboxylic acids in 6‐maleimidocaproic acid, a preparatory modification of the latter is necessary, as shown in Figure 1b. The reaction of 6‐maleimidocaproic acid with oxalyl chloride yields 6‐maleimidocaproyl chloride, which is then employed to modify lignin. The infrared spectrum in Figure 2d distinctly shows enhanced absorption peaks for C ═ O bonds and the characteristic maleimide rings.^[^ ^22^ ^]^ The ^1^H NMR spectra of lignin and LMA exhibit significant differences. A signal corresponding to the hydrogen atoms on the diene group appears at 6.99 ppm, while signals from the hydrogen atoms on the alkyl chain are observed between 1 and 2 ppm (Figure 2e).^[^ ^29^ ^]^ The ^13^C NMR spectrum also shows a strong carbonyl signal, and distinct signals corresponding to each carbon atom in the maleimide group are clearly identified (Figure S3, Supporting Information).^[^ ^29^ ^]^ As shown in the survey spectra (Figure S4, Supporting Information), a pronounced N1s signal emerged in the LMA sample, which is absent in unmodified lignin, clearly indicating the introduction of nitrogen‐containing groups. High‐resolution XPS analysis of the LMA sample revealed two N 1s peaks at 399.89 and 401.53 eV (Figure 2f). The former is attributed to C─N single bonds, potentially arising from minor ring‐opening or lignin interactions, while the latter corresponds to nitrogen atoms within the maleimide ring, with its elevated binding energy resulting from conjugation effects and ring strain. These features collectively confirm the successful incorporation and structural integrity of maleimide groups in LMA.

### 2.2 Optical and Thermal Properties of CAF‐L Film

**Figure 3:**
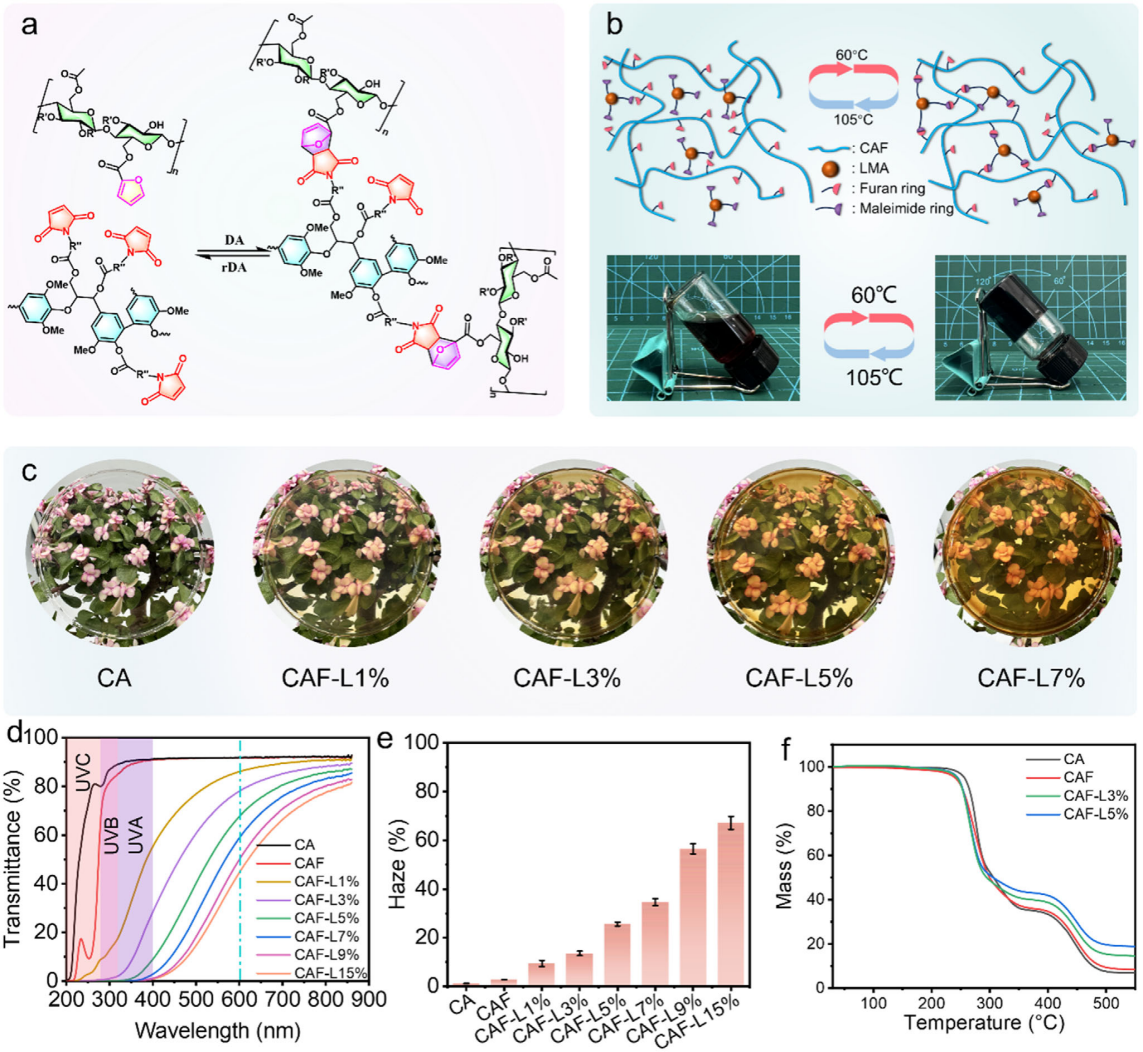
a) The chemical equation and b) schematic diagram of the Diels‐Alder reaction between CAF and LMA, along with a visual photograph of the DA reaction conducted in DMSO. c) High‐resolution digital images of CA and CAF‐L films with varying LMA contents. d) UV–vis transmittance spectra and e) haze data of CA and CAF‐L films with different LMA contents. f) TG curves for CA, CAF, CAF‐L3%, and CAF‐L5%.

The Diels‐Alder reaction, a dynamic covalent reaction that is sensitive to temperature,^[^ ^30^ ^]^ involves two components: dienes and dienophiles,^[^ ^31^ ^]^ which correspond to the furan and maleimide groups grafted onto cellulose acetate and lignin, respectively. The prepared CAF and LMA are mixed in specified ratios and cast into films through solvent evaporation, forming CAF‐L films. These films are then heat‐treated under varying temperatures and durations to achieve Diels‐Alder cross‐linked CAF‐L‐H films. As supported by previous reports and our macroscopic observations (**Figure** **3**a,b), the temperature range of 40–90 °C primarily favors the forward DA reaction, while 100–120 °C favors the retro‐DA reaction.^[^ ^32^, ^33^, ^34^, ^35^, ^36^ ^]^ It is important to note that both reactions occur simultaneously in dynamic equilibrium, with the equilibrium position being temperature‐dependent. To visually illustrate the thermal reversibility of DA bonds, a homogeneous solution of CAF and LMA in DMSO was alternately placed in ovens preheated to 60 °C and 105 °C. The mixture formed a translucent gel at 60 °C and returned to a clear liquid at 105 °C within several minutes, demonstrating the sol‐gel‐sol transition driven by reversible DA bond formation and cleavage in solution. During heat‐treatment above the glass transition temperature, the enhanced chain mobility of cellulose acetate increases the exposure of pendant furan groups, thus facilitating more efficient cross‐linking with maleimide groups on LMA. In this process, LMA acts as an intermediary that promotes cross‐linking within the polymer network. This cross‐linking alters the microstructure of the films, subsequently impacting their macroscopic properties. Infrared spectroscopy of the CAF‐L films before and after heat‐treatment (Figure S5, Supporting Information) shows a decrease in the absorption peaks of C═C bonds and furan rings, confirming the occurrence of the Diels‐Alder reaction.

As shown in Figure 3c, digital photographs of CAF‐L films with varying LMA mass fractions demonstrate a gradual darkening in color as the LMA content increases. To further improve the visibility of these differences, we have included additional photographs taken on a white background in Figure S6 (Supporting Information), which more clearly highlight the reduction in transparency and increase in color intensity across samples. The UV–visible transmittance spectra of CAF‐L films, depicted in Figure 3d, utilize transmittance at 600 nm as an indicator of film transparency.^[^ ^37^ ^]^ As the LMA content increases from 1% to 15%, the transmittance decreases progressively from 86.1% to 44.5%, indicating reduced transparency. This trend is consistent with the visual appearance of the films and can be attributed to the increasing concentration of conjugated aromatic structures and phenolic chromophores introduced by LMA, which effectively absorb both UV and visible light. In particular, the CAF‐L films exhibit strong UV‐shielding capabilities across the UVA (320–400 nm), UVB (280–320 nm), and UVC (200–280 nm) regions, owing to the abundant UV‐absorbing functional groups in LMA.^[^ ^38^ ^]^ These spectral changes, together with visual observations, confirm the light‐attenuating role of lignin derivatives in the composite films. As shown in Figure 3e, haze values also increase significantly with LMA content. CA and CAF films maintain low haze values (<2%), while the CAF‐L films show haze increasing from 9% to 67% as LMA increases from 1% to 15%. This haze increase can be attributed to enhanced light scattering caused by LMA molecular aggregation or microphase domain formation, which reduces clarity and enhances opacity. Notably, when the LMA weight percentage exceeds 9%, LMA macromolecules aggregate, significantly increasing the film's haze (Figure S7, Supporting Information). This phenomenon is likely attributed to the enhanced intermolecular interactions between LMA chains, such as π–π stacking and hydrogen bonding, which promote local aggregation and increase light scattering within the film. Surface examinations of CA, CAF‐L3%, CAF‐L5%, and CAF‐L7% via SEM and their corresponding EDS analyses (Figure S8, Supporting Information) indicate smooth, dense surfaces devoid of cracks and large particles. EDS scans reveal a uniform distribution of LMA within the films, with nitrogen content positively correlating with the LMA mass fraction.

The TGA and DTG profiles of CAF‐L, as illustrated in Figure 3f and Figure S10 (Supporting Information), indicate that CA begins to lose mass at 220 °C, with the rate of mass loss peaking at 275 °C. The rate diminishes and stabilizes ≈355 °C, primarily attributed to the cleavage of ester bonds within CA, breakdown of the cellulose backbone, and liberation of acetyl groups.^[^ ^39^ ^]^ Upon further heating to 390 °C, CA undergoes subsequent mass loss phases, initiating carbonization until complete decomposition. Compared to unmodified CA, the modified CAF exhibits an earlier onset of mass loss, albeit with a reduced rate of thermal decomposition, thereby demonstrating comparable thermal stability. The introduction of LMA does not significantly alter the initial thermal degradation temperature of the films, which remains approximately at 210 °C, similar to that of CAF. As the LMA mass fraction increases, the residual char content also rises. This is primarily due to the increased LMA content and the stabilizing effect of its aromatic structures, which promote the formation of a stable char layer. To further clarify the thermal behavior of the maleimide‐modified lignin component, the TGA curve of LMA alone was provided (Figure S11, Supporting Information). The results showed that LMA exhibited an initial decomposition temperature ≈215 °C, followed by a major weight loss stage between 250–400 °C, corresponding to the breakdown of the aliphatic chains and maleimide groups. A substantial char residue remained above 450 °C, attributed to the aromatic‐rich lignin backbone. These results confirm the excellent thermal stability of LMA and support its role in improving the char yield and thermal resistance of CAF‐L composite films. Additionally, surface contact angle tests were conducted, showing that CA had a static contact angle which increased after the introduction of the furan ring in CAF, and further increased upon the addition of LMA, reaching a peak of 111° before gradually decreasing to 80° (Figure S12, Supporting Information). The increased contact angle is attributed to enhanced surface roughness caused by LMA, which exposed some LMA particles on the film surface. The phenolic hydroxyl groups on lignin, not fully substituted during the LMA modification, enhanced the surface free energy, leading to the observed decrease in contact angle.

### 2.3 Tunable Mechanical Properties of CAF‐L Film through Heat‐treatment

To investigate the impact of dynamic Diels‐Alder bonds on the mechanical properties of CAF‐L films, we conducted mechanical performance tests before and after heat‐treatment, focusing on variations in LMA content and different heat‐treatment conditions. The presence of furan and maleimide groups within the films, forming dynamic Diels‐Alder bonds, significantly affects their stress‐strain behavior. By adjusting heat‐treatment conditions, the cross‐linking density is modulated, enabling fine‐tuning of mechanical properties such as elasticity and fracture toughness.^[^ ^40^ ^]^ Moreover, given the dynamic nature of DA bonds, the internal network structure may continue to rearrange even after film formation or heat‐treatment. To reduce this effect and ensure comparability across samples, all films were stored in a desiccator at room temperature for 24 h before and after thermal treatment to allow partial equilibration prior to testing.

**Figure 4:**
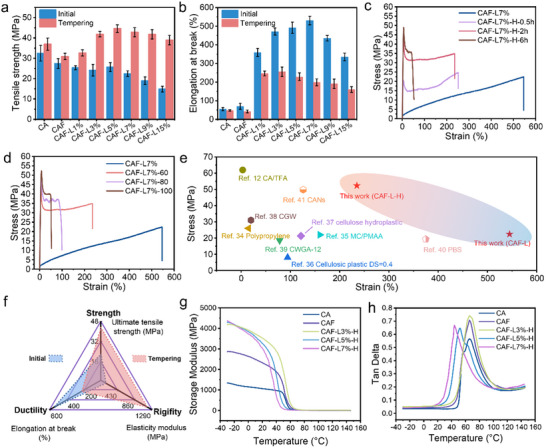
a) Tensile strength bar chart, and b) elongation at break bar chart for CA and CAF‐L films with varying LMA contents, before and after heat‐treatment at 60 °C for 2 h. c) Stress‐strain curves for CAF‐L7% films under initial conditions and heat‐treated at 60 °C for 0.5, 2, and 6 h. d) Stress‐strain curves for CAF‐L7% films under initial conditions and heat‐treated at 60 °C, 80 °C, and 100 °C for 2 h. e) Summarize the previously reported mechanical properties of various materials.^[^ ^12^, ^41^, ^42^, ^43^, ^44^, ^45^, ^46^, ^47^, ^48^ ^]^ f) Radar chart comparing the mechanical performance of CAF‐L films before and after heat‐treatment. g) Storage modulus and h) Tan delta data from DMA testing of samples after heat‐treatment (60 °C, 2 h).

The stress and strain of both CA and CAF exhibited no significant changes before and after heat‐treatment, confirming that the heat‐treatment process does not affect the fundamental properties of the films (Figure S13, Supporting Information). Prior to heat‐treatment, the films exhibited relatively low crosslinking density between dynamic Diels‐Alder bonds in CAF and LMA, resulting in lower fracture stress and higher elongation at break, indicative of substantial flexibility. The stress‐strain curves of CAF‐L demonstrate characteristics typical of amorphous polymers.^[^ ^49^ ^]^ Before heat‐treatment, LMA acts as a ‘plasticizer’ between polymer chains, facilitating their slippage and rearrangement under stress, thereby significantly enhancing the elongation at break.^[^ ^50^, ^51^ ^]^ This ease of molecular chain slippage makes yield points difficult to discern, resulting in a smooth transition from elastic to plastic deformation. Additionally, the incorporation of LMA does not indefinitely enhance the toughness of CAF‐L; excessive “plasticizer” can impede molecular slippage, leading to macroscopic film rupture as the LMA mass fraction increases, with the elongation at break initially peaking at 530% before declining. Heat‐treatment promotes the formation of dynamic Diels‐Alder bonds and cross‐linking between CAF and LMA, transitioning the stress‐strain characteristics from an amorphous profile to one exhibiting clear yielding behavior (**Figure** **4**a,b).^[^ ^52^ ^]^ The tensile strength of CAF‐L‐H notably increases to a maximum of 44.6 MPa, a 95% increase from pre‐heat‐treatment levels. This enhancement in tensile strength is primarily attributed to the formation of dynamic Diels‐Alder bonds and intermolecular cross‐linking.^[^ ^53^ ^]^ Heat‐treatment alters the orientation of molecular chains and enhances intermolecular interactions, thereby augmenting the material's load distribution capabilities and structural stability. As cross‐link density increases, the overall ductility of CAF‐L‐H decreases, consequently reducing the elongation at break. For CAF‐L7%, the elongation at break decreases from 530% to 199% after heat‐treatment. This is attributed to cross‐linking points restricting molecular mobility, thereby reducing material plasticity. With increasing LMA content, this trend is further intensified, leading to a continued decline in ductility.

Heat‐treatment conditions significantly impact the mechanical properties of the films, as illustrated in Figure 4c and Figure S14 (Supporting Information), which displays the stress‐strain curves for CAF‐L3%, 7%, and 9% at a consistent temperature of 60 °C across varying heat‐treatment durations, and for a fixed duration of 2 h at different temperatures. It is evident from the graphs that at a constant temperature, as the heat‐treatment duration increases, the fracture stress of the films significantly rises, while the elongation at break decreases, with the highest fracture stress reaching 49.1 MPa and the elongation at break reducing to 52.9%. At the same duration, varying the temperature also markedly alters the mechanical performance of CAF‐L. For instance, at 100 °C for 2 h, the fracture stress peaks at 52.3 MPa and the elongation at break drops to 52.7% (Figure 4d; Figure S15, Supporting Information). These phenomena indicate changes in the internal structure of the material during heat‐treatment, particularly an increase in cross‐link density, which enables the material to withstand greater external forces without fracturing.^[^ ^54^ ^]^ Heat‐treatment shifts the equilibrium of the DA reaction toward adduct formation within an optimal temperature range, increasing the instantaneous concentration of crosslinked bonds and thereby enhancing the mechanical strength of the material.^[^ ^55^ ^]^ Concurrently, this increased cross‐linking reduces the material's ductility, as more cross‐link points restrict the mobility of molecular chains, thereby diminishing the material's extensibility under stress. The dynamic adjustment of heat‐treatment temperature and time provides crucial experimental evidence for designing and manufacturing high‐performance bio‐based materials with specific mechanical properties. It is worth noting that under moderate thermal conditions (≈60 °C), the CAF‐L network may exhibit slight viscoelastic relaxation due to increased segmental mobility near its glass transition temperature; however, the continued slow formation of DA bonds at this temperature contributes to progressive network consolidation, allowing the material to retain sufficient structural integrity while defining a practical thermal boundary for stable mechanical performance. To further evaluate the mechanical performance of CAF‐L, its tensile strength and elongation at break were compared with other biomass‐based plastics (Figure 4e). Both CAF‐L and CAF‐L‐H films exhibited exceptional performance in terms of tensile strength and elongation at break, demonstrating excellent mechanical tunability. Notably, after heat‐treatment, the CAF‐L‐H films, through the incorporation of more dynamic covalent crosslinks, showed enhanced mechanical strength and ductility. This process also improved the material's recyclability and processability, significantly increasing its potential for widespread application in practical settings. As illustrated in Figure 4f, a radar chart highlights the mechanical adaptability of the system by visually comparing tensile strength and elongation at break. The distinct profiles of CAF‐L and CAF‐L‐H reflect their respective mechanical emphases, with CAF‐L favoring higher ductility while CAF‐L‐H exhibits greater strength. This confirms that the mechanical behavior of the films can be programmably adjusted to meet specific performance requirements.

To investigate the thermomechanical properties of the materials, dynamic mechanical analysis (DMA) was performed on samples after heat‐treatment at 60 °C for 2 h, as shown in Figure 4g,h and Figure S16 (Supporting Information). The incorporation of furan rings and LMA significantly influenced the mechanical properties compared to pure CA. The results show a marked decrease in the storage modulus (E') with increasing temperature, demonstrating the materials' thermosoftening behavior. However, the modified films exhibited higher initial storage moduli. At −30 °C, the storage modulus of CA was 1.3 GPa, while that of CAF increased to 2.9 GPa. CAF‐L films, depending on the LMA content, showed even higher values, ranging from 4.1 to 4.3 GPa. This increase in E′ observed below Tg is primarily attributed to the complex aromatic structure and high molecular weight of lignin, which restricts the mobility of polymer chains.^[^ ^56^ ^]^ Additionally, dynamic Diels‐Alder bonds formed between the furan rings and maleimide groups further enhance material rigidity at lower temperatures, with the bonds becoming reversible at higher temperatures. This interpretation is further supported by DMA data of unheated CAF‐L films (Figure S17, Supporting Information), which exhibited a lower storage modulus and a slightly reduced Tg compared to heat‐treated samples, confirming the increase in crosslink density upon thermal activation. The sharp decline in storage modulus ≈50–60 °C corresponds to the glass transition of the polymer network. The loss modulus initially increases and then decreases, peaking near Tg, reflecting the maximum internal friction and viscoelastic energy dissipation, consistent with the energy dissipation observed during the glass transition of polymers. Compared to CA, CAF and CAF‐L films exhibit higher loss moduli, indicating superior energy dissipation capabilities, which can be attributed to the dynamic Diels‐Alder bonds formed.^[^ ^57^ ^]^ The loss factor (tan δ), which characterizes the material's viscoelasticity and internal damping performance, typically indicates Tg.^[^ ^58^ ^]^ In this study, the tan δ peak shifts to lower temperatures with increasing LMA content, which at first appears to suggest a decrease in Tg. However, this can be better understood as a manifestation of network heterogeneity: although rigid lignin segments enhance E′ at low temperatures, the dynamic covalent network introduces local plasticization, leading to broader and slightly lower tan δ peaks. This change reflects an increase in structural rigidity, which limits the mobility of molecular segments and reduces the energy dissipation and damping performance near Tg. Overall, the incorporation of furan rings and LMA enhances the mechanical strength and thermal stability of cellulose acetate. While LMA increases structural rigidity, it also moderately reduces the damping performance at elevated temperatures.

To directly verify the occurrence of the DA reaction within the CAF‐L system, Differential Scanning Calorimetry (DSC) was performed on CAF, CAF‐L5%, and CAF‐L7% films. As shown in Figure S18 (Supporting Information), pure CAF displayed only a subtle baseline shift near 60 °C, indicating a typical glass transition without significant thermal events. In contrast, both CAF‐L5% and CAF‐L7% exhibited a mild exothermic peak in the 50–65 °C range and a clear endothermic peak between 125–135 °C. The low‐temperature exothermic peak corresponds to the forward DA reaction, in which furan and maleimide groups form thermally stable covalent linkages. The higher‐temperature endothermic event is consistent with the retro‐DA reaction, in which these dynamic bonds undergo dissociation.^[^ ^59^ ^]^ These two distinct thermal signatures strongly indicate the reversible nature of DA chemistry in our system and provide compelling evidence that crosslinking via DA reaction occurs progressively under mild thermal conditions. This reversible thermal behavior was further confirmed by multi‐cycle DSC tests showing reproducible exo‐/endothermic transitions over multiple heating scans (Figure S18d, Supporting Information). This thermally triggered behavior confirms the dynamic covalent nature of the polymer network and supports our design of tunable properties through heat treatment. Notably, the onset of the forward DA reaction overlaps with the glass transition temperature of the matrix. This indicates that as polymer chains gain mobility during Tg, more furan and maleimide groups become accessible, thereby facilitating enhanced crosslinking efficiency at relatively low thermal input. To further verify the thermal stability of the dynamic crosslinked network, temperature‐sweep rotational rheometry was performed (Figure S19, Supporting Information). The storage modulus remained consistently higher than the loss modulus across the entire heating range, confirming that the material maintained a solid‐like elastic state during heating. These findings collectively demonstrate that the DA network is thermally stable under moderate processing temperatures and supports the tunability of mechanical properties through heat treatment. We further quantified the thermal signature of DA crosslinking by integrating the exothermic peak observed in the DSC curve of CAF‐L5%. A reaction enthalpy of ≈−2.42 J/g was obtained over the 40–75 °C range. This small value is consistent with partial and progressive DA bond formation in a spatially constrained polymer matrix, and supports the concept of reversible thermally triggered crosslinking that underpins our material design.^[^ ^60^, ^61^, ^62^ ^]^

**Figure 5:**
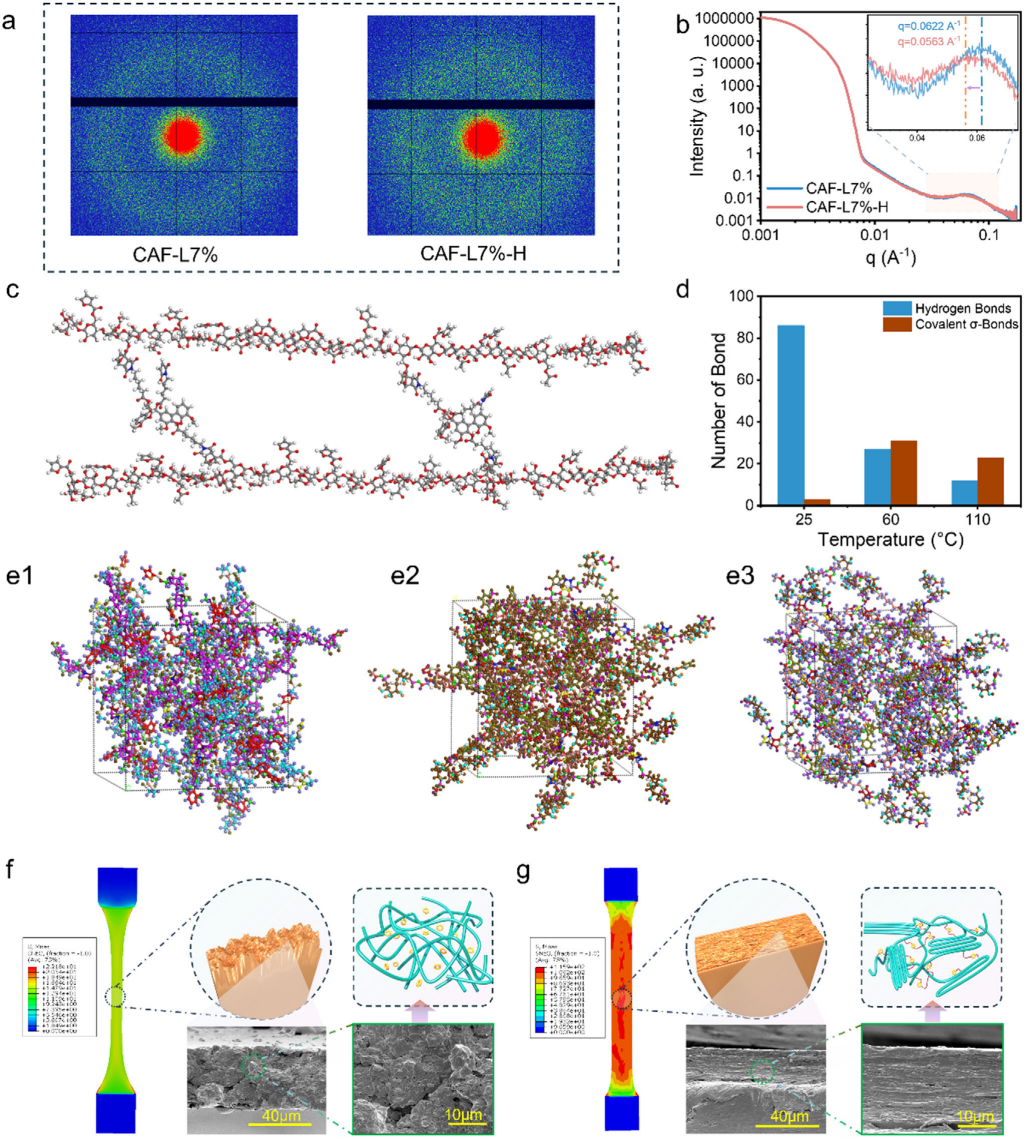
a) 2D small‐angle X‐ray scattering (SAXS) patterns of CAF‐L7% and CAF‐L7%‐H. b) SAXS profiles of CAF‐L7% and CAF‐L7%‐H. c) Macroscopic crosslinking simulation diagram of CAF and LMA in molecular dynamics. (d) The number of hydrogen bonds and covalent σ‐bonds in the CAF‐L network at different temperatures. Structural evolution in molecular dynamics simulations, showing molecular conformations at (e1) 25 °C, (e2) 60 °C, and (e3) 110 °C. Finite element simulated pre‐fracture stress distribution, post‐fracture SEM morphology, and molecular arrangement schematic of CAF‐L‐7% film g) before and h) after heat‐treatment.

To unravel the underlying structural basis for the pronounced mechanical reinforcement observed in heat‐treated CAF‐L films, a comprehensive characterization combining small‐angle X‐ray scattering (SAXS), molecular dynamics (MD) simulations, bond evolution analysis, and fracture surface morphology was conducted. The 2D SAXS patterns (**Figure** **5**a) exhibit isotropic halos in both untreated and heat‐treated samples, with no evidence of lamellar ordering or crystalline alignment. Notably, the halo in the annealed CAF‐L7%‐H sample appears slightly larger and more diffuse, indicating a shift toward lower q‐values. This expansion of the scattering ring suggests an increase in average domain spacing, which can be attributed to thermally induced chain relaxation and local structural loosening.^[^ ^63^ ^]^ Such rearrangement is consistent with the behavior of dynamic covalent networks and implies greater mobility and flexibility within the polymer matrix. The SAXS profiles (Figure 5b) reveal a notable shift in the characteristic scattering peak from q = 0.0622 Å⁻¹ to q = 0.0563 Å⁻¹, corresponding to an increase in domain spacing from 10.10 nm to 11.16 nm. This expansion suggests that heat‐treatment induces mesoscopic structural reorganization, likely driven by increased chain mobility and the partial reversibility of Diels‐Alder linkages. The enlarged domain spacing reflects polymer chain relaxation and enables more frequent interactions between reactive groups, thereby facilitating the formation of additional covalent cross‐links. As a result, the internal network becomes less densely packed yet more interconnected and structurally adaptive. This mesoscopic reorganization of the polymer network, facilitated by thermally triggered chain mobility and dynamic Diels‐Alder linkages, indicates a shift in internal architecture toward a more compliant yet interconnected structure.^[^ ^64^ ^]^ In fact, thermally activated Diels–Alder crosslinking promotes the formation of reversible covalent bonds, resulting in a network that is more interconnected at the molecular level. This network refinement reduces free volume and disrupts diffusion pathways, leading to improved barrier performance.^[^ ^59^ ^]^

To gain insight into the temperature‐responsive crosslinking mechanism of the CAF‐L dynamic covalent network, molecular dynamics simulations were performed based on a 3D model constructed from CAF and LMA. The simulations analyzed the evolution of hydrogen bonds and σ‐bonds across different temperatures, as well as their impact on network morphology. Figure 5c illustrates the macroscopic architecture of the CAF‐L network, formed by Diels‐Alder reactions between furan groups on CAF and maleimide groups on LMA, generating thermally reversible σ‐bonds. This architecture establishes a structurally continuous and thermally responsive framework. The molecular dynamics optimized models of CAF and LMA, along with a schematic illustration of their micro‐scale crosslinking process, are presented in Figure S20 (Supporting Information). Figure 5e1‐e2 depicts representative structural snapshots at 25 °C, 60 °C, and 110 °C, respectively, showing how temperature modulates interchain interactions and spatial organization. At 25 °C (Figure 5e1), the network is loosely entangled and primarily stabilized by hydrogen bonds, with sparse covalent crosslinks. At 60 °C (Figure 5e2), DA reactions are strongly activated, resulting in a surge in σ‐bond formation and a corresponding decline in hydrogen bonding. The network becomes more densely packed, with enhanced chain alignment and restricted segmental mobility. At 110 °C (Figure 5e3), retro‐Diels–Alder reactions are triggered, increasing σ‐bond dissociation events. Hydrogen bonding remains low, and chain relaxation occurs, indicating the onset of structural reconfiguration and partial network deconstruction. To quantify these effects, we analyzed the number of hydrogen and σ‐bonds across the three temperatures (Figure 5d). Hydrogen bond count decreases steadily throughout the heating process, reflecting the thermal instability of weak intermolecular forces.^[^ ^65^ ^]^ In contrast, σ‐bond formation peaks at 60 °C and subsequently declines at 110 °C, suggesting a dynamic equilibrium between forward and reverse DA reactions. This interplay defines a three‐phase thermal pathway that involves hydrogen bond‐dominated entanglement in the initial stage, followed by the formation of dynamic covalent crosslinks, and ultimately leads to reversible network reconfiguration. These findings provide direct molecular evidence of how the CAF‐L network transitions from a weakly associated structure into a densely cross‐linked, thermally adaptable material. The synergistic modulation of hydrogen bonding and dynamic covalent interactions underpins the system's mechanical enhancement and structural tunability.^[^ ^66^ ^]^

This thermally modulated network, governed by the cooperative evolution of dynamic Diels‐Alder bonds and hydrogen bonding, results in a densely cross‐linked and spatially continuous framework in CAF‐L. The resulting changes in chain mobility and stress transmission pathways provide a direct structural basis for the observed mechanical transformation.^[^ ^66^ ^]^ To further explore how this network architecture governs macroscopic mechanical behavior, finite element simulations were conducted to analyze stress distribution and failure mechanisms before and after heat treatment. The results are presented in Figure 5f,g, Figures S21, S22 (Supporting Information). Among them, Figure S21 (Supporting Information) compares the simulated stress‐strain curves with the experimentally measured tensile curves. The results demonstrate that not only do the two curves exhibit a high degree of similarity in overall shape, but they also show strong agreement in key mechanical parameters, such as the yield point, peak stress, and fracture strain, with numerical deviations maintained within a reasonable range. This indicates that the established finite element model possesses a high predictive capability for the actual mechanical response of the material under tensile loading. At the initial stage of tensile deformation, the stress distribution in CAF‐L remains relatively uniform, primarily concentrated in the central region of the specimen. As deformation progresses, stress gradually propagates along the axial direction, and even just before failure, the specimen maintains a relatively homogeneous strain distribution. This suggests that the molecular chains within CAF‐L have high mobility, allowing significant chain slippage under stress, which contributes to the material's greater ductility. However, due to the lack of sufficient cross‐linking points, the maximum stress it can withstand is relatively low, resulting in lower tensile strength. After heat‐treatment, the internal Diels‐Alder cross‐linking network in CAF‐L‐H is significantly strengthened, leading to a notable change in the stress distribution characteristics. During loading, stress concentration regions in CAF‐L‐H increase markedly, particularly near the gripping ends and the central region of the specimen. This indicates that the cross‐linked network enhances intermolecular interactions, increasing the overall rigidity of the material. However, this strengthening effect also promotes earlier localized failure, making the fracture behavior more brittle, which aligns with the experimentally observed trend of increased strength but reduced elongation.

The SEM fracture surface images (including CAF‐L‐3% and CAF‐L‐5%) further confirm these findings (Figure 5f,g; Figure S23, Supporting Information). In CAF‐L, the fracture surface is relatively rough, exhibiting clear signs of plastic deformation. This confirms that the material undergoes significant molecular chain slippage during stretching, contributing to its high ductility. CAF‐L primarily consists of a loosely arranged molecular chain network with a low degree of cross‐linking, resulting in a softer mechanical profile. In contrast, CAF‐L‐H exhibits a more layered fracture morphology, with SEM observations revealing a denser and smoother fracture surface. This indicates that the cross‐linked network restricts molecular mobility, leading to a more brittle fracture mode. Additionally, heat‐treatment induces the Diels‐Alder cross‐linking reaction and facilitates molecular chain rearrangement, enhancing the structural uniformity and mechanical strength of the material. These microstructural rearrangements are further supported by AFM analysis (Figure S24, Supporting Information), which reveals that the surface of CAF‐L7% prior to heat‐treatment exhibits pronounced striped undulations with a height difference of 245.5 nm, suggesting localized stress aggregation and loose chain packing. After heat‐treatment, the surface becomes more homogeneous and less rough (213.8 nm), reflecting enhanced chain entanglement and network densification due to dynamic crosslinking. This conclusion is further supported by the transformation of the stress–strain curve after heat‐treatment into a characteristic crystalline polymer tensile curve. Such significant microstructural changes are attributed to the formation of dynamic Diels‐Alder bonds during heat‐treatment, which reinforces intermolecular interactions, thereby significantly increasing the fracture stress while reducing the fracture elongation. These observations, along with the increased tensile strength and reduced elongation at break, confirm that heat‐induced DA crosslinking restricts chain rearrangement and plastic deformation. Such a ductile‐to‐brittle transition mechanism has also been reported in other thermally crosslinked polymer networks.^[^ ^67^ ^]^

### 2.4 Practical Formability and Gas Barrier Properties of CAF‐L Film

**Figure 6:**
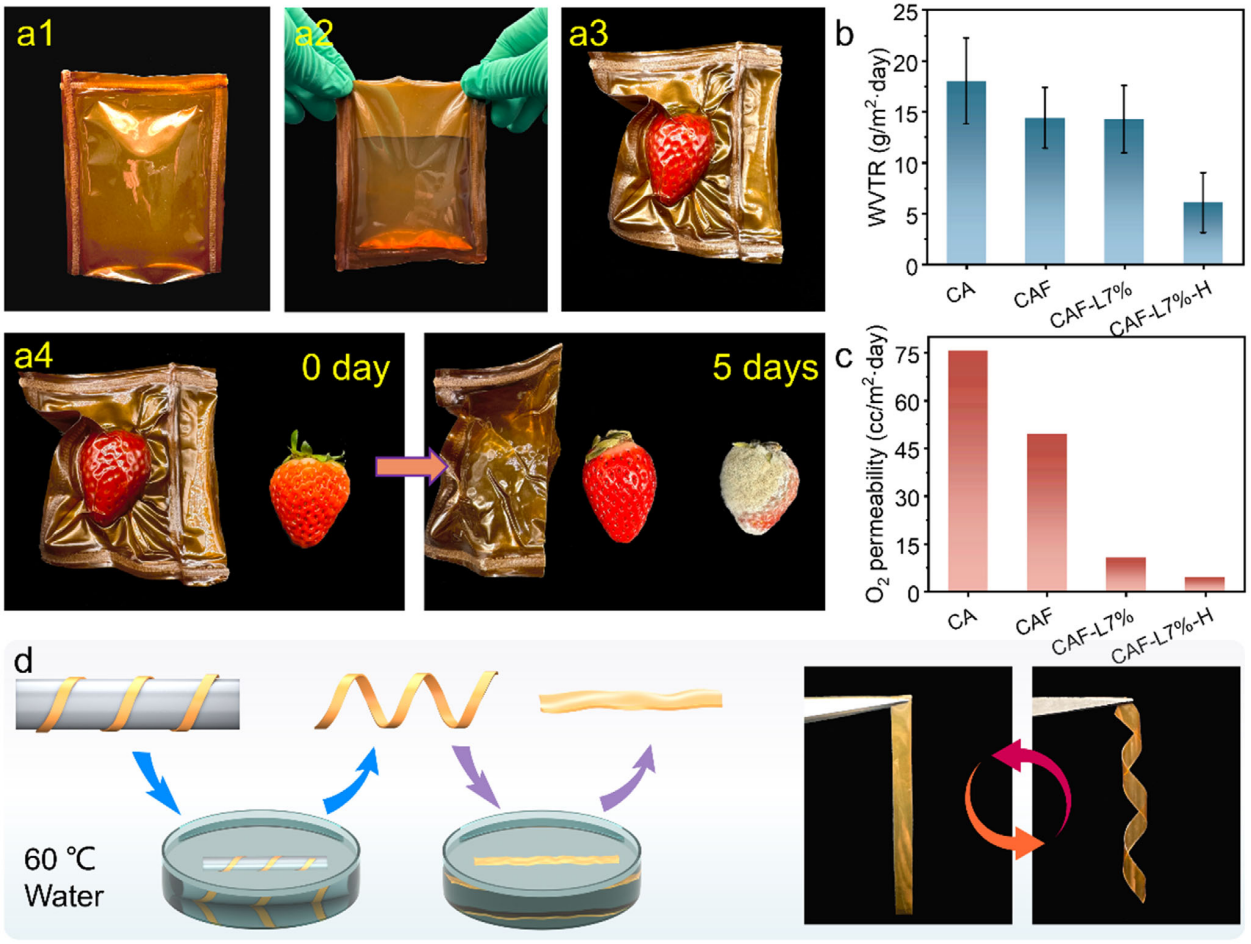
a1) Packaging bags made from CAF‐L via heat sealing, followed by a2) liquid containment testing and a3) vacuum sealing fruit testing, a4) digital photographs showing the condition of strawberries after 5 days, packaged in CAF‐L versus exposed to air. b) Water vapor permeability and c) oxygen permeability data for CA, CAF, CAF‐L7%, and CAF‐L7%‐H films. d) Schematic diagram and digital photo of CAF‐L films, which retain specific shapes after immersion in hot water at 60 °C for 30 s and recover their shape upon re‐immersion.

The multifunctionality and application potential of CAF‐L materials have been comprehensively demonstrated, particularly in the fields of packaging and recycling. As shown in **Figure** **6**a, 1, CAF‐L films can be processed into various shapes using heat sealing, with the heat seal strength significantly higher than that of conventional CA materials, increasing from 24.4 to 36.6 MPa, representing an enhancement of ≈50% (Figure S25, Supporting Information). This substantial performance improvement is primarily attributed to the introduction of dynamic Diels‐Alder bonds, which effectively enhance the structural integrity and load‐bearing capacity of the material.^[^ ^59^ ^]^ Due to its excellent hydrophobicity and mechanical properties, the heat‐sealed CAF‐L films are capable of effectively containing liquids and performing vacuum sealing of fruits (Figure 6a2,a3). For example, strawberries sealed in CAF‐L bags remained fresh for 5 days at room temperature, with no signs of decay, while those exposed to air rotted and developed mold (Figure 6a4). This clearly demonstrates the superior food preservation properties of CAF‐L.

In practical applications, the gas permeability tests are essential for evaluating material properties.^[^ ^68^ ^]^ In this study, we assessed the water vapor transmission rate (WVTR) and oxygen permeability (O_2_ permeability) of CA, CAF, CAF‐L7% and CAF‐L7%‐H (Figure 6b,c). The results indicated that unmodified CA, due to its relatively loose molecular structure, exhibited higher WVTR and O_2_ Permeability, thus facilitating greater penetration by water vapor and oxygen. After modification of CA with furfuryl chloride, both WVTR and O2 Permeability significantly decreased, suggesting that the newly formed cross‐links enhanced the structural density and thus improved barrier properties.^[^ ^69^ ^]^ For the CAF‐L7% sample, the heat‐treatment (CAF‐L7%‐H) further reduced the WVTR, and O_2_ Permeability also showed notable improvements, confirming that heating facilitated the cross‐linking of dynamic Diels‐Alder bonds, thereby enhancing the overall structural stability and barrier properties of the material. By managing the chemical modification and subsequent heat‐treatment processes, the gas and water vapor barrier properties of polymeric materials can be effectively regulated, which is crucial for designing high‐performance polymer applications with specific barrier requirements. Notably, films with a 5% mass fraction of LMA displayed the highest contact angles, correlating with the WVTR data, indicating that CAF‐L7% maintained high water vapor barrier properties while also exhibiting relatively high static contact angles. Furthermore, the modified CAF‐L films still retains thermoplastic properties and can be shaped in hot water at 60 °C into various forms, such as spirals, rings, rectangles, and stars, and retains these shapes upon cooling (Figure 6d; Figure S26, Supporting Information). Notably, CAF‐L exhibits shape memory characteristics, quickly returning to its original form after repeated immersion in hot water or heating (Videos S1 and S2, Supporting Information).

### 2.5 Recyclability of CAF‐L Film

**Figure 7:**
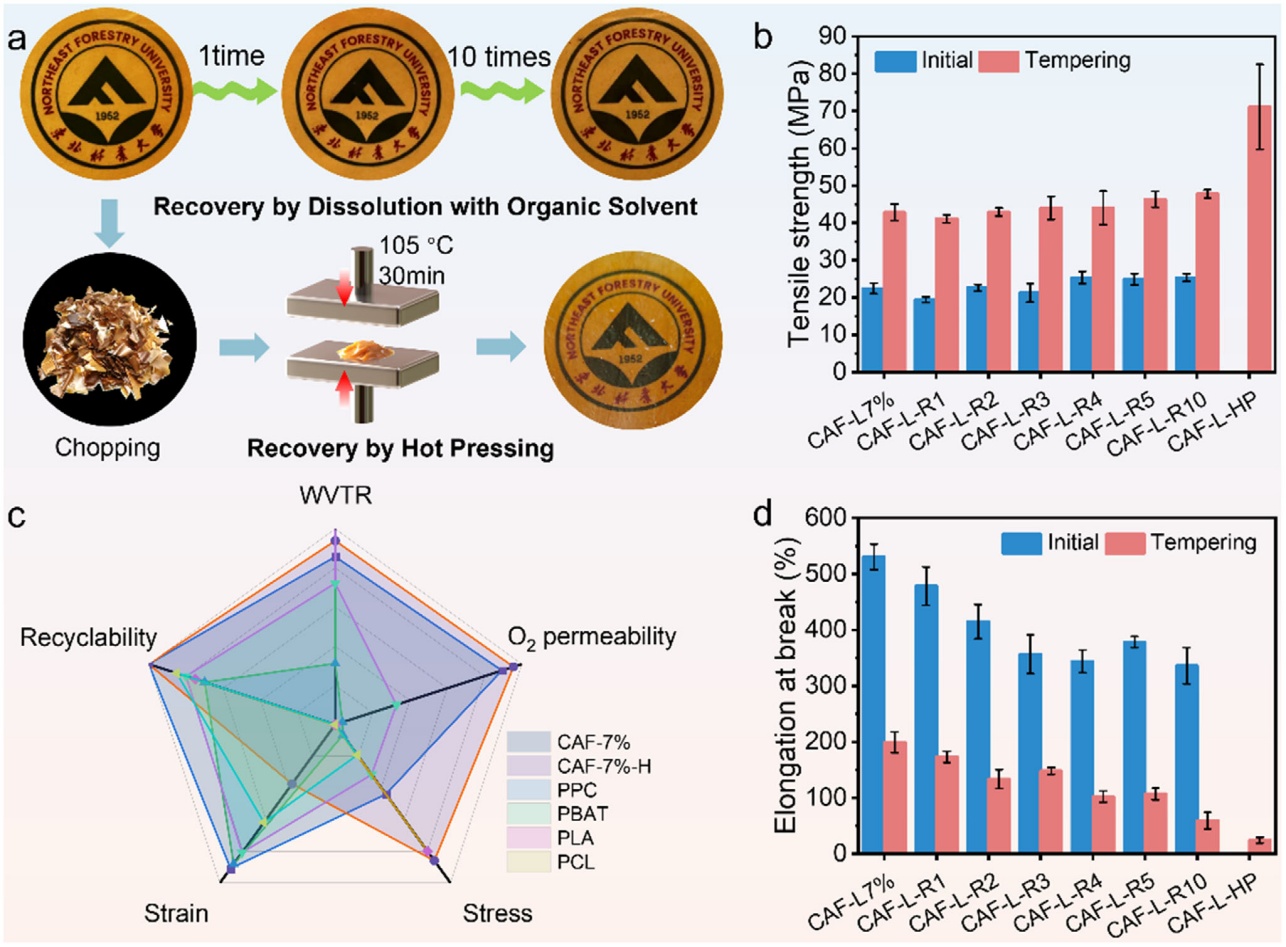
a) Digital photographs and schematic illustration of the CAF‐L‐7% recycling and recyclability process: CAF‐L films were recycled either by dissolution in an organic solvent or by hot pressing at 105 °C for 30 min after being chopped. b) Tensile strength and d) elongation at break of CAF‐L‐7% films before and after multiple recycling cycles (CAF‐L‐R1 to CAF‐L‐R10) and hot‐pressing recovery (CAF‐L‐HP). c) Radar chart comparing the mechanical properties, gas barrier performance, and recyclability of CAF‐L films with representative commercial bio‐based plastics.^[^ ^77^, ^78^, ^79^, ^80^ ^]^

Due to the presence of dynamic covalent bonds, CAF‐L films maintain excellent recyclability while enhancing mechanical properties.^[^ ^70^ ^]^ They can completely dissolve and regenerate in 5 min when soaked in DMSO at 105 °C. However, without heating, the films retain their complete form even after being immersed in DMSO for 48 h, showing no signs of dissolution (Figure S27, Supporting Information). We also evaluated the stability of CAF‐L films in various solvents (Figure S28, Supporting Information), common solvents caused only swelling, not complete dissolution. Recyclability tests were conducted on CAF‐L7% through 10 cycles of dissolution and recyclability, demonstrating that the films preserved their original appearance (**Figure** **7**a) and showed no significant change in tensile strength (Figure 7b), maintaining a strength of 47.7 MPa after 10 cycles. However, the elongation at break decreased to 336% after these cycles (Figure 7d). The primary reason for the change in mechanical properties after recycling is the gradual shortening of the cellulose molecular chains and the alteration in the scale of intermolecular sliding, leading to reduced elongation at break.^[^ ^71^, ^72^, ^73^ ^]^ Conversely, the shortening of molecular chains facilitates increased Diels‐Alder reactions, contributing to the enhancement of tensile strength.^[^ ^74^ ^]^ In addition to solvent‐based recycling, hot pressing was employed as an alternative recovery method to evaluate its ability to maintain mechanical integrity while offering a more sustainable and solvent‐free recyclability process with reduced chemical waste. As illustrated in Figure 7a, chopped CAF‐L film fragments were subjected to hot pressing at 105 °C for 30 min, enabling the reformation of Diels‐Alder bonds and restoring the material's structural integrity. Notably, after hot pressing, the tensile strength of the regenerated films exhibited a remarkable improvement, reaching ≈75 MPa (Figure 7b). This value significantly surpasses that of the dissolution‐recovered samples and even exceeds the tensile strength of the initially heat‐treated CAF‐L films, indicating that the applied thermal and compressive conditions facilitated enhanced cross‐linking density and molecular rearrangement.^[^ ^75^ ^]^ Figure 7c presents a radar chart comparing CAF‐L and CAF‐L‐H with typical biodegradable polymers—poly(propylene carbonate) (PPC), poly(butylene adipate‐co‐terephthalate) (PBAT), poly(lactic acid) (PLA), and poly(ε‐caprolactone) (PCL)—across five key dimensions including tensile stress, elongation, oxygen permeability, water vapor transmission rate (WVTR), and recyclability. The CAF‐L‐H film displayed a balanced and superior profile, particularly in strength, barrier properties, and recycling performance, underscoring the comprehensive advantages of dynamic covalent networks over conventional bio‐based plastics.^[^ ^76^ ^]^

## 3 Conclusion

This work presents a scalable and sustainable strategy for fabricating high‐performance bio‐based films through Diels‐Alder dynamic covalent crosslinking between furfuryl‐functionalized cellulose acetate and maleimide‐modified lignin. By employing heat‐treatment to activate the DA reaction, we achieved programmable modulation of mechanical properties, enabling smooth transitions between high strength and high ductility states. The films exhibit excellent recyclability through both solvent dissolution and hot pressing, with both methods preserving mechanical integrity and demonstrating structural reconfigurability. Moreover, enhanced UV shielding, gas barrier capability, and shape retention further extend the functional applicability of CAF‐L films. Overall, this study provides a promising platform for designing mechanically tunable, environmentally adaptable, and fully bio‐based polymeric materials, offering a viable alternative to petroleum‐derived plastics in sustainable packaging and circular economy applications.

## 4 Experimental Section

### Materials and Chemicals

Cellulose acetate (acetyl content: 32.0 wt.%, hydroxyl content: 8.7 wt.%, degree of substitution: 1.75, esterification value: 44.6) was purchased from Macklin (China). Dealkalized lignin (Aladdin, China) was used with the following specifications: ignition residue (as sulfate): 10–25%, methoxyl content (calculated on anhydrous basis): 10–12.5%, and moisture content (Karl Fischer): 0–20%. Furfuryl chloride (97%), oxalyl chloride (98%), and 6‐maleimidohexanoic acid (98%) were also obtained from Aladdin (China). Tetrahydrofuran (THF, 99.5%), anhydrous ethanol (EtOH), N,N‐dimethylacetamide (DMAc, 99.7%), N,N‐dimethylformamide (DMF, 99.7%), and dichloromethane (DCM, 99.5%) were all analytical grade and supplied by Tianjin Fuyu Fine Chemicals Co., Ltd. All chemicals and reagents were used as received without further purification.

### Preparation of Cellulose Acetate Furanoate (CAF)

1g of cellulose acetate was dried in an oven at 60 °C for 6 h and then dissolved in 30 mL of DMAc. The solution was heated to 50 °C in an oil bath, and 0.68 mL of furfuryl chloride (1 mol of hydroxyl group corresponds to 1 mol of furfuryl chloride) was added under stirring. After reacting for 4 h at 50 °C, the solution was poured into 200 mL of ethanol to precipitate, followed by centrifugation. The precipitate was retained and dissolved in THF, followed by precipitation in ethanol, and the process was repeated to remove excess reactants. The product was vacuum dried for 12 h and stored in THF for further use.

### Preparation of Maleimide‐Modified Lignin (LMA)

The synthesis of LMA was carried out in two steps. First, 6‐maleimidohexanoic acid (1.0 g, 4.6 mmol) was dissolved in 5 mL of DCM, and oxalyl chloride (1.0 mL, 11.5 mmol) was added dropwise under magnetic stirring at room temperature. The reaction proceeded for 1 h until gas evolution ceased, indicating complete conversion to the corresponding acyl chloride, which was used directly without further purification.

Subsequently, 1.0 g of dealkalized lignin was dispersed in 30 mL of DMF and stirred at 50 °C. The freshly prepared acyl chloride was added dropwise, and the mixture was allowed to react at 50 °C for 4 h. After completion, the reaction mixture was cooled to room temperature, and deionized water was added. The pH was adjusted to ≈4 using 10% (v/v) HCl, resulting in the precipitation of the modified lignin. The precipitate was collected by centrifugation (5000 rpm, 10 min), thoroughly washed with ethanol and deionized water, and finally redissolved in THF for storage.

### Preparation of Diels‐Alder Cross‐linked CAF‐L Film

CAF and LMA were mixed in THF at varying LMA mass fractions (1 wt%, 3 wt%, 5 wt%, 7 wt%, 9 wt%, and 15 wt% relative to the mass of CAF). For a representative formulation, 42 mg of LMA and 600 mg of CAF were dissolved in 15 mL of THF and subjected to ultrasonication for 20 min to ensure homogeneous mixing. The resulting uniform solution was cast into a flat‐bottomed glass Petri dish (9 cm in diameter) and allowed to evaporate at ambient conditions (25 ± 2 °C, relative humidity 45–55%) for 24 h. The formed CAF‐L film was then peeled off and vacuum‐dried for 12 h to remove residual solvent.

To induce Diels‐Alder crosslinking, the dried CAF‐L films were subjected to heat treatment at different temperatures (60 °C, 80 °C, and 100 °C) for varying durations (0.5 h, 2 h, and 6 h). All heat treatments were conducted in a temperature‐controlled air‐circulating oven to ensure uniform thermal exposure and reproducibility.

### Recycling Tests of CAF‐L

1 g of CAF‐L film was heated to 105 °C and maintained at this temperature for 30 min to induce the cleavage of dynamic Diels‐Alder bonds. The film was then placed in tetrahydrofuran and vigorously shaken until it completely dissolved. The resulting solution was poured into a flat‐bottomed petri dish, and the CAF‐L‐R film was obtained by evaporating the solvent at room temperature.

1 g of CAF‐L film was chopped into small fragments and hot‐pressed at 105 °C under 20 MPa for 30 min. The temperature was then gradually lowered to 60 °C and maintained for 1 h to facilitate structural stabilization, yielding the regenerated CAF‐L‐HP film.

### Molecular Dynamics Simulation

To elucidate the temperature‐dependent dynamics of the Diels–Alder and retro‐Diels–Alder reactions within the CAF‐L polymeric network, all‐atom molecular dynamics (MD) simulations were performed using Materials Studio 2020. Amorphous cell models of CAF and LMA chains were first constructed using the AC module to simulate disordered polymer morphology. Geometry optimization was conducted using the Smart Minimizer algorithm, and subsequent MD calculations were performed using the Forcite module under the COMPASS II force field. Periodic boundary conditions were applied in all 3D to mimic bulk behavior. Atomic partial charges were automatically assigned based on force field parameters. Temperature and pressure were controlled using the Andersen thermostat and Berendsen barostat, respectively. The cutoff radius for van der Waals and electrostatic interactions was set to 15.5 Å. To simulate the temperature‐governed progression of DA and rDA reactions, the polymer system was subjected to a three‐stage thermal protocol: 10‐cycle annealing to achieve initial relaxation; equilibrium simulations at 298, 333, and 383 K (representing ambient temperature, DA‐favorable temperature, and rDA‐favorable temperature, respectively); extraction of representative conformations.

### Characterization

The chemical structures of CAF and LMA were characterized by liquid‐state ¹H and ¹^3^C NMR spectroscopy using a Bruker AVANCE III 400 MHz NMR spectrometer (deuterated solvents: DMSO‐d₆ for CA, CAF, Lignin, and LMA), X‐ray photoelectron spectroscopy (Thermo Scientific K‐Alpha), and FTIR spectroscopy (Nicolette 6700, Thermo Fisher Scientific, MA, USA). The cross‐sectional morphology of the samples was characterized using a scanning electron microscope (Merlin Compact, Carl Zeiss AG, Germany). The elemental composition and distribution of the samples were further studied using EDS (X‐Max‐20mm^2^, Oxford Instruments, UK) in combination with SEM. Water contact angles were measured at room temperature using a contact angle goniometer (Attension Theta, Biolin Scientific, Sweden). Differential scanning calorimetry (DSC) was conducted using a TA Q2000 instrument. Approximately 5–8 mg of sample was sealed in an aluminum pan. Samples were initially heated from 20 °C to 160 °C at a rate of 20 °C min^−1^ and held isothermally for 10 min to erase previous thermal history and promote dissociation of reversible Diels–Alder bonds. After cooling to 20 °C, the samples were reheated to 160 °C at 2 °C min^−1^, and all thermal transitions, including glass transition, exothermic and endothermic peaks, were obtained from the second heating curve. Thermogravimetric analysis (TG 209 F1, China) was conducted under an Ar atmosphere at a heating rate of 10°C min^−1^ from 30°C to 550°C. Dynamic rheological measurements were performed using an AR2000ex rotary rheometer (TA Instruments, USA) equipped with a parallel‐plate geometry (diameter: 25 mm, gap: 1.0 mm), with temperature sweep tests conducted in oscillatory mode from 20 °C to 150 °C at a constant frequency of 1 Hz and a heating rate of 5 °C min^−1^. The room temperature mechanical properties of the composites were measured using a universal testing machine (C41.103, XinSanSi Enterprise Development Co. Ltd., China). During testing, the samples were cut into regular rectangles (40 mm × 10 mm) with an initial gauge length of 20 mm and a tensile speed of 5 mm min^−1^. The results were averaged based on five samples. Prior to testing, all specimens were stored in a vacuum desiccator at room temperature for 24 h to allow partial structural equilibration. The dynamic mechanical properties of the samples were analyzed utilizing a dynamic mechanical analyzer (TA, Q800, USA). Measurements were conducted in tension mode at an oscillation frequency of 1 Hz, with a temperature ramp from −30 °C to 150 °C at a rate of 5 °C min^−1^. Atomic force microscopy (AFM) was performed using a Multimode 8 system (Bruker, USA) in tapping mode to observe the fracture surface morphology of tensile‐tested films. Small‐angle X‐ray scattering (SAXS) measurements were performed using a Xenocs Xeuss 2.0 system (France) equipped with a Cu Kα source (λ = 0.154 nm). The system operated at a tube power of 30 W, with a focal spot diameter of 30 µm and a maximum photon flux at the sample position of 4.5 × 10⁸ photons/s. Scattered X‐rays were collected using a Dectris Eiger2 R 1M detector with a pixel size of 75 µm.

## Conflict of Interest

The authors declare no conflict of interest.

## Supporting information



Supporting Information

Supplemental Video 1

Supplemental Video 2

## Data Availability

The data that support the findings of this study are available from the corresponding author upon reasonable request.
