# Supplementary material for: Cellulosic Functional Bioplastic with Tunable Strength and Toughness Through Heat‐Treatment of Dynamic Covalent Networks
Source: Adv Sci (Weinh). 2025 Jul 21;12(38):e08075. doi: 10.1002/advs.202508075 (PMC12520483; doi:10.1002/advs.202508075)
Supplement: Supplementary file 1 — Supporting Information [file ADVS-12-e08075-s002.docx]

Supporting Information

**Cellulosic Functional Bioplastic with Tunable Strength and Toughness through Heat-treatment of Dynamic Covalent Networks**

Xiangyu Tang, ^a^ Linlin Zhao, ^a^ Yunfeng Guo, ^a^ Ying Wang, ^a^ Zhenke Wei, ^a^ Xinyan Fan, ^a^ Zefang Xiao, ^a^ Haigang Wang, ^a^ Yanjun Xie, ^a^ Yonggui Wang, ^a,^ *


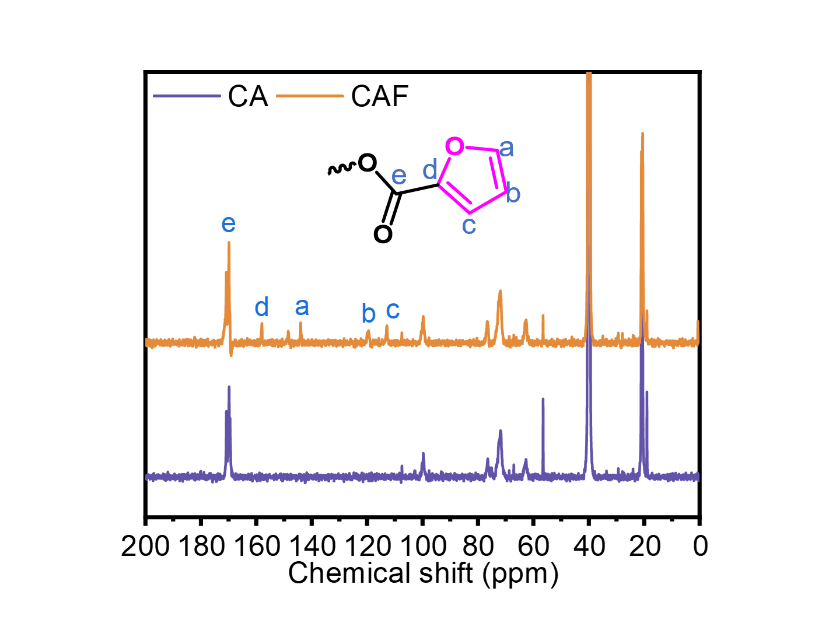


Figure S1. ^13^C NMR spectra of CA and CAF.


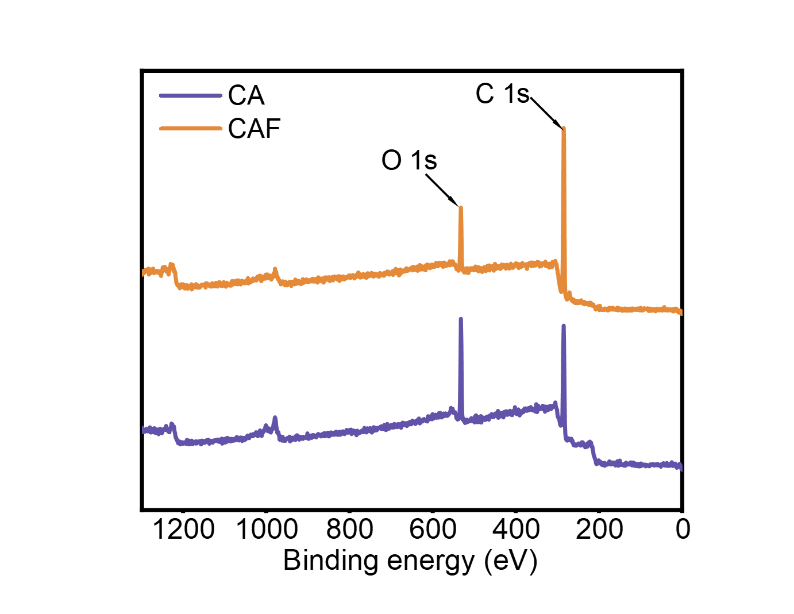


Figure S2. Full XPS spectra of CA and CAF.


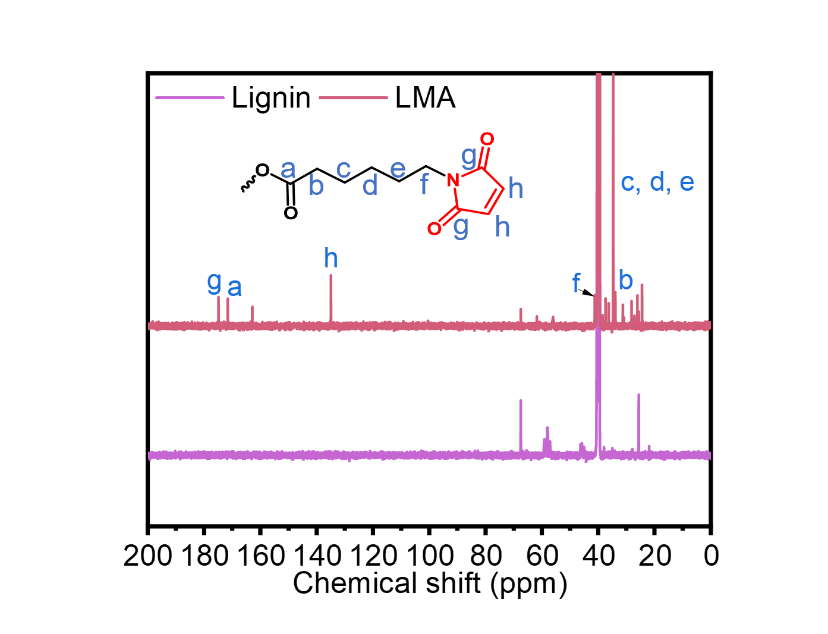


Figure S3. ^13^C NMR spectra of Lignin and LMA.


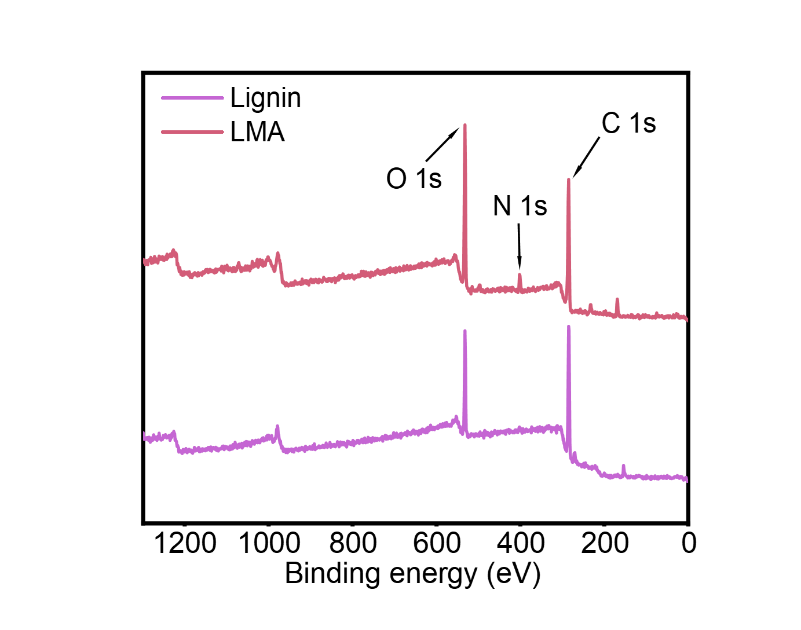


Figure S4. Full XPS spectra of Lignin and LMA


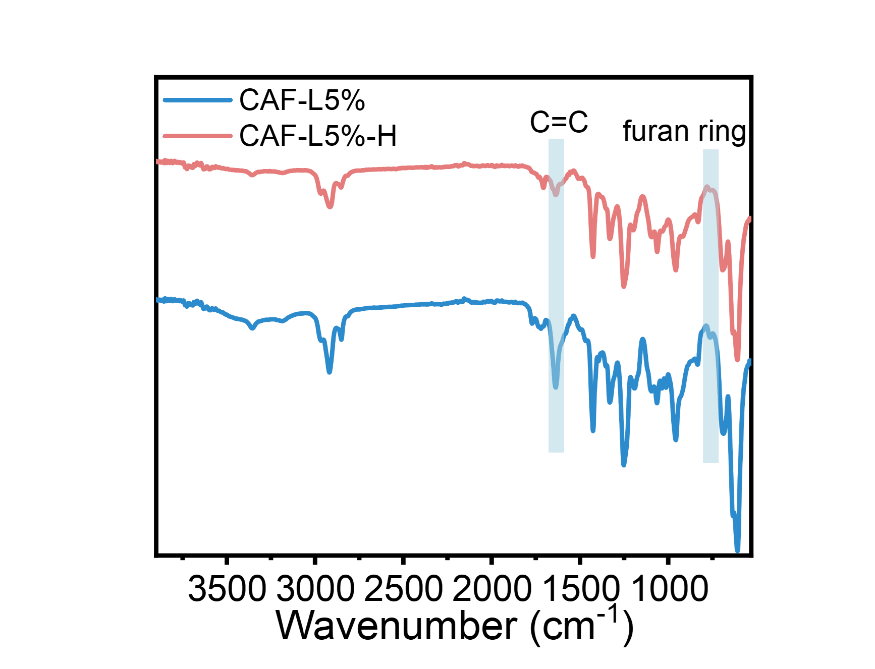


Figure S5. FTIR spectra of CAF-L5% before and after heat-treatment.


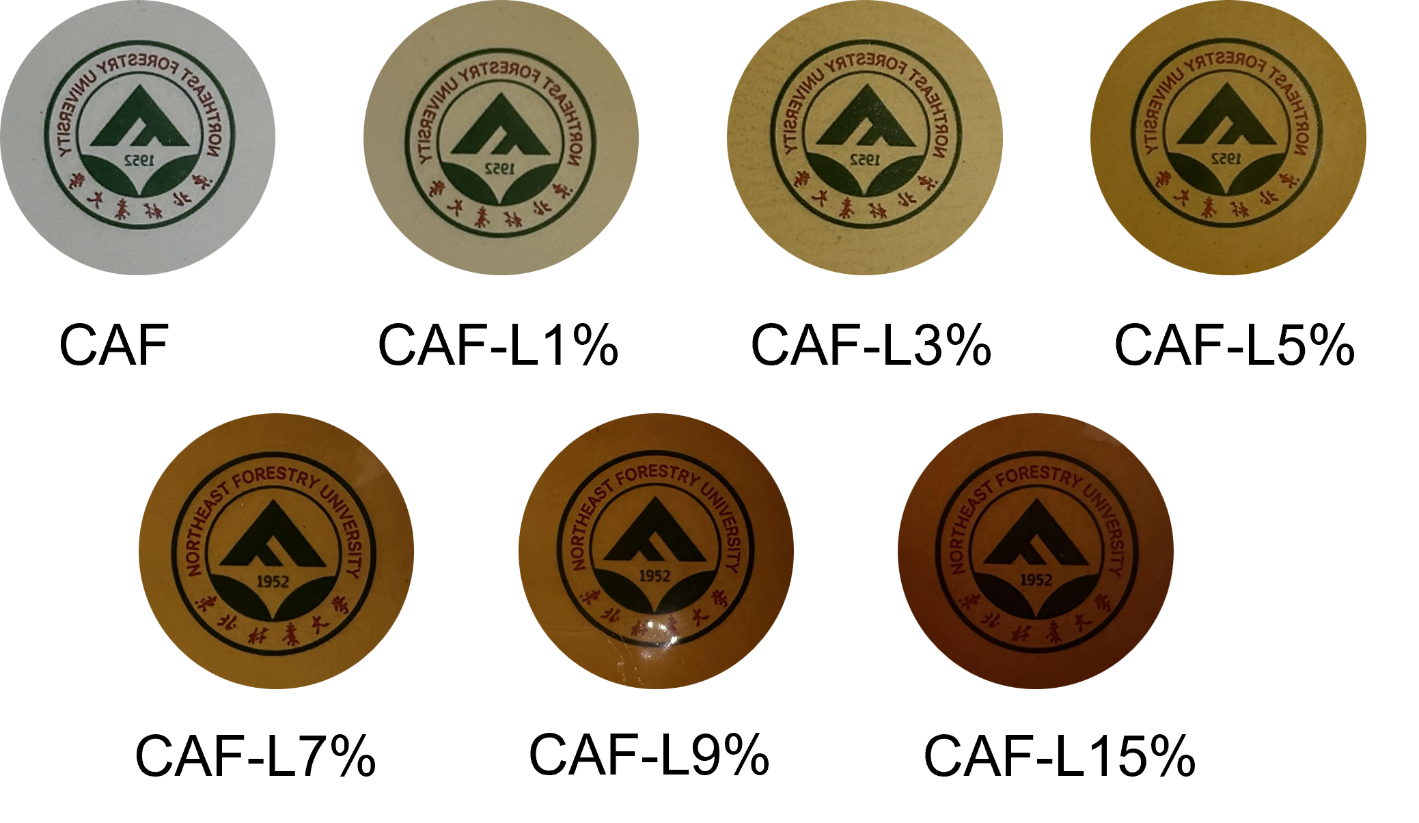


Figure S6. Photographs of CAF-L films with increasing LMA content placed on white background.


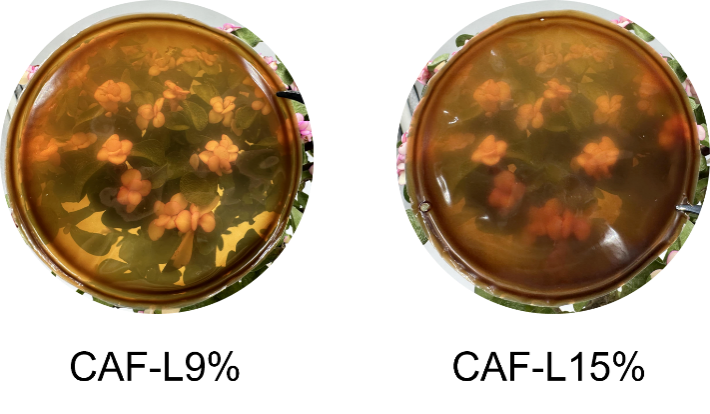


Figure S7. Digital photographs of CAF-L9%, and CAF-L15%.


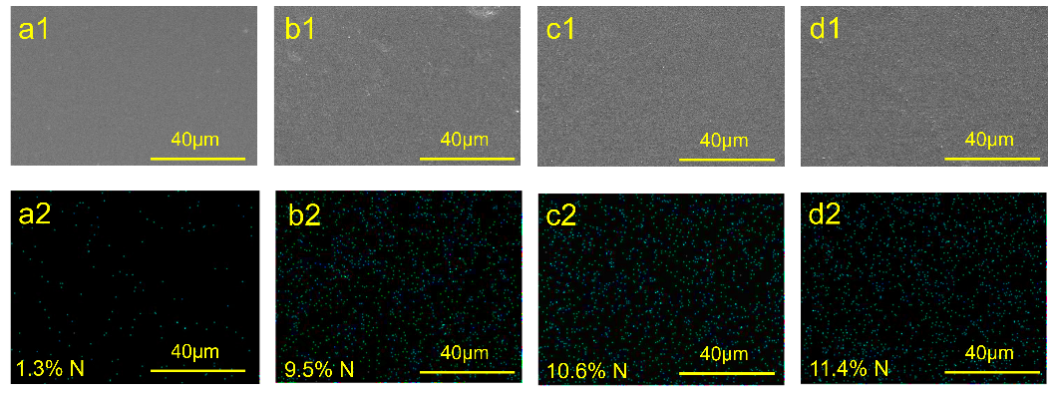


Figure S8. SEM surface images and surface EDS maps showing nitrogen distribution for (a1) CA, (a2) CAF-L3%, (a3) CAF-L5%, and (a4) CAF-L7%.


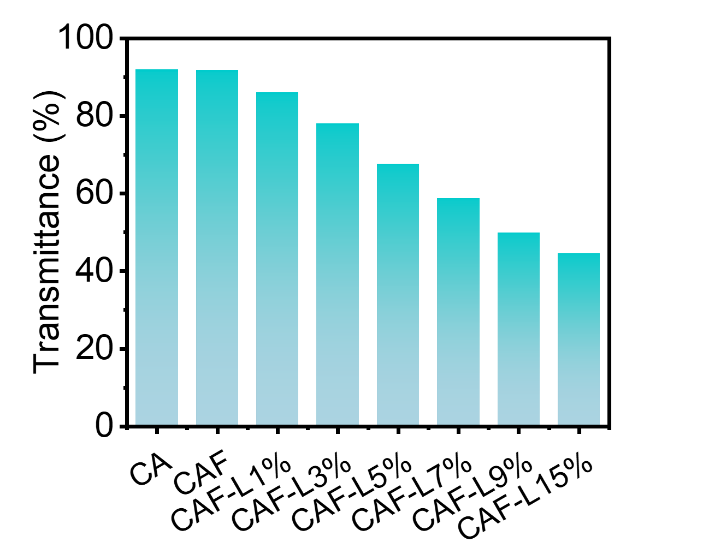


Figure S9. Optical transmittance at 600 nm for CA and CAF-L with varying LMA contents.


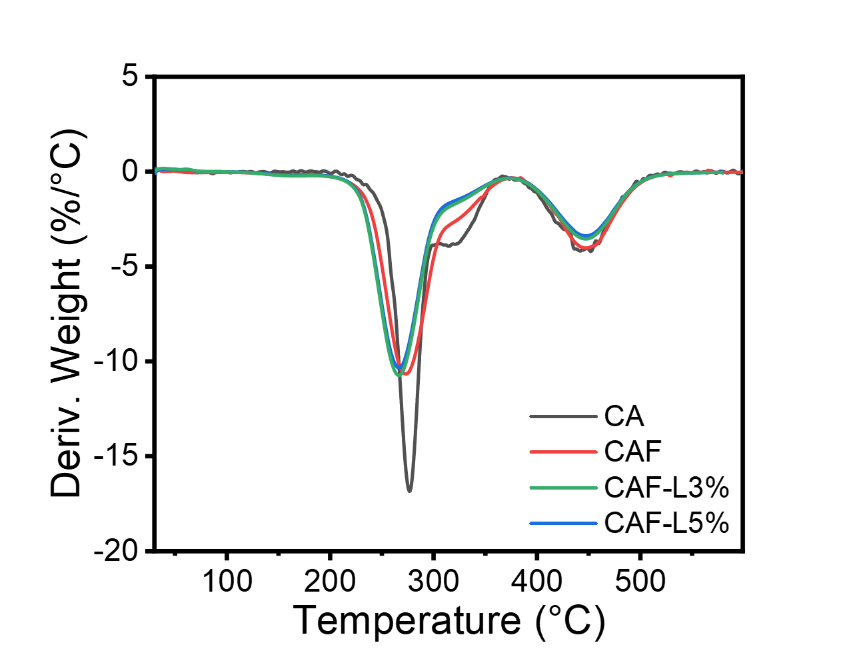


Figure S10. DTG curves for CA, CAF, CAF-L3%, and CAF-L5%.


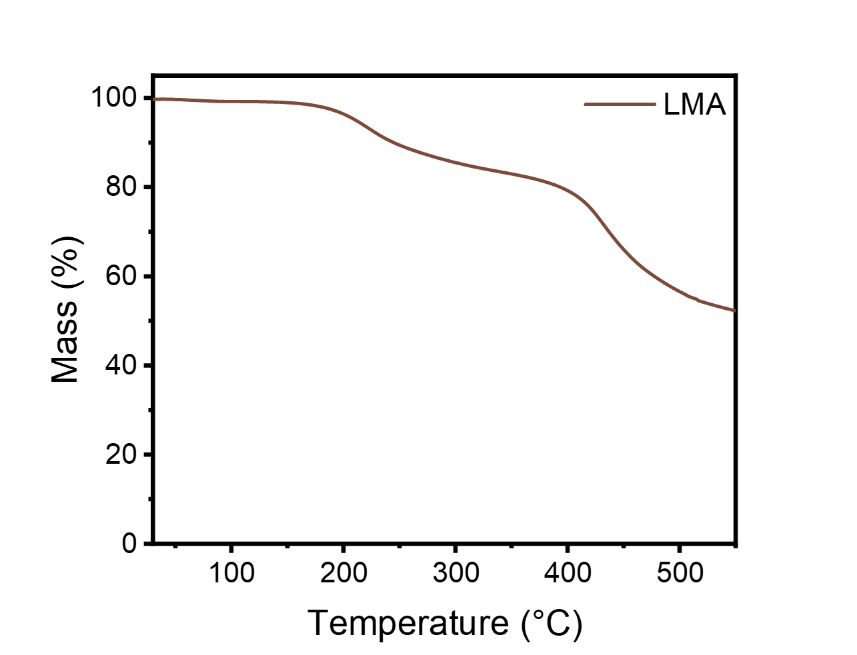


Figure S11. TG curves for LMA.


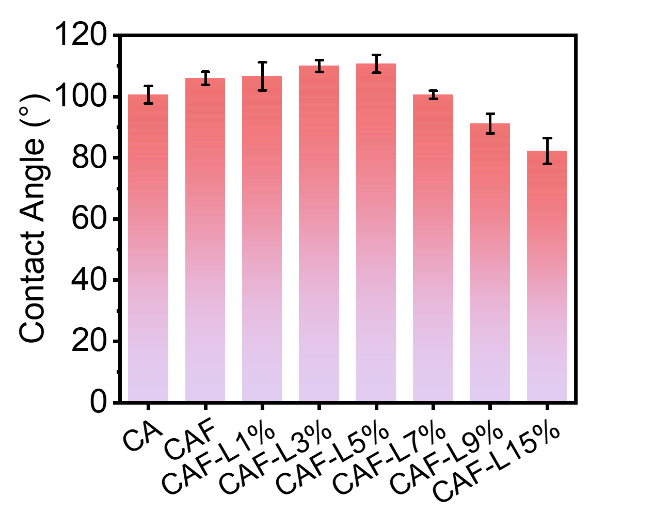


Figure S12. Contact angle data for CA and CAF-L films with varying LMA contents.


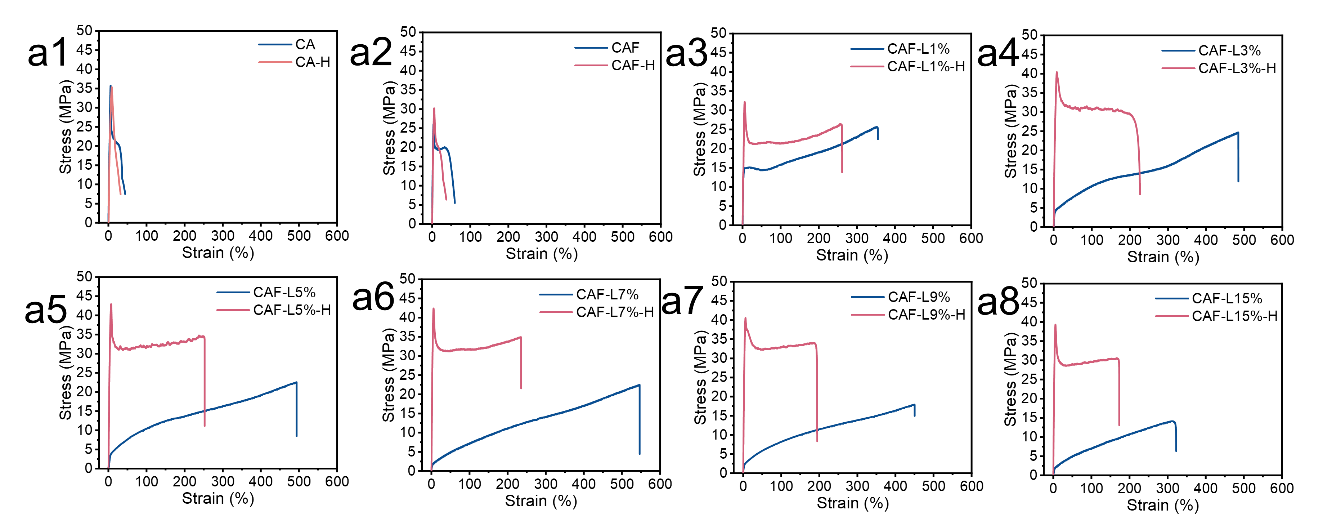


Figure S13. Stress-strain curves for CA and CAF-L films with varying LMA contents before and after heat-treatment at 60 °C.


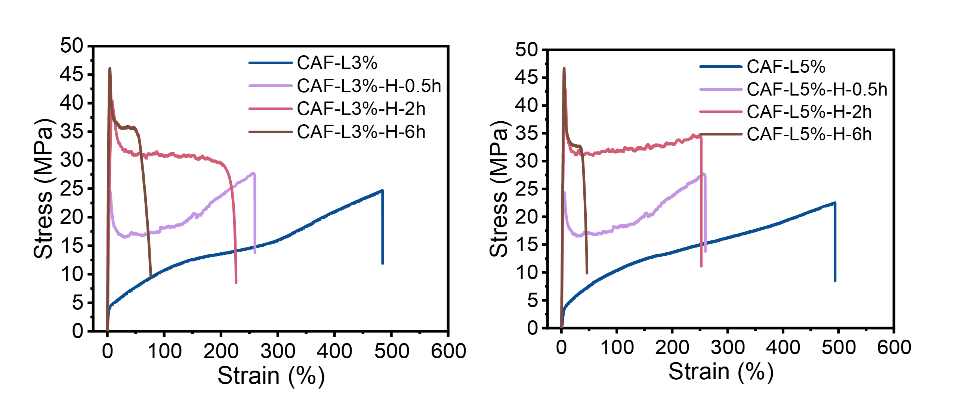


Figure S14. Stress-strain curves for CAF-L3% and CAF-L5% initially and after heat-treatment at 60 °C for 0.5h, 2h, and 6h.


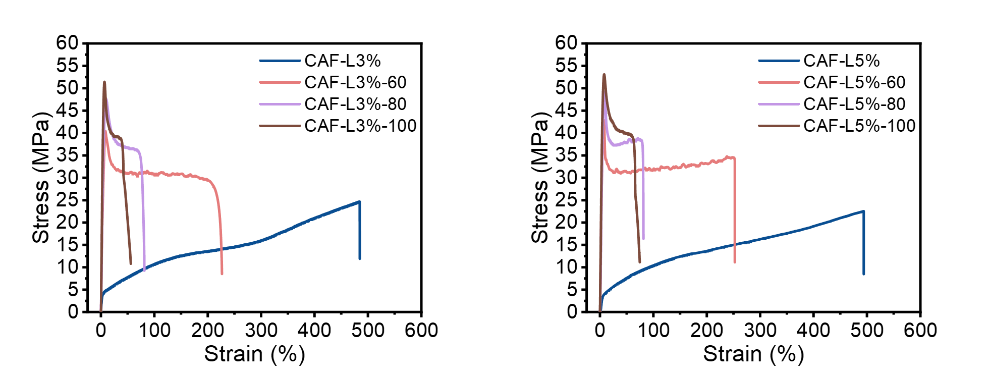


Figure S15. Stress-strain curves for CAF-L3% and CAF-L5% initially and after 2 hours of heat-treatment at temperatures of 60 °C, 80°C, and 100°C.


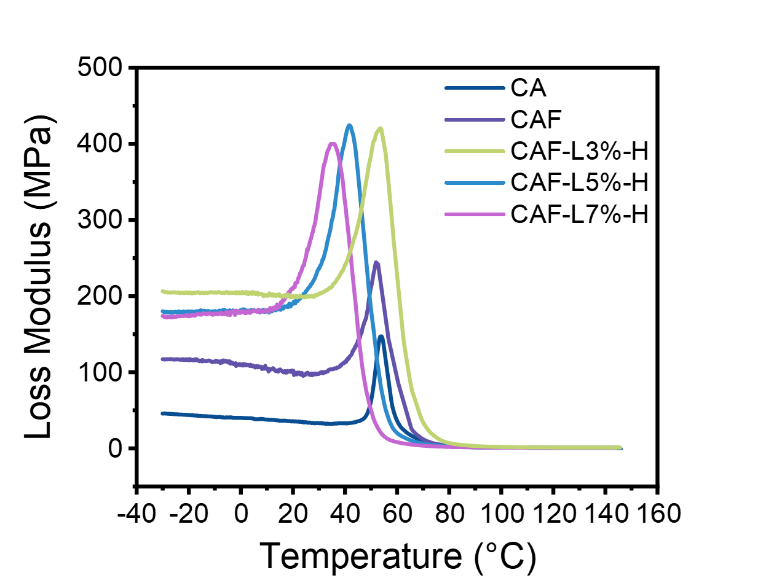


Figure S16. Loss modulus from DMA testing.


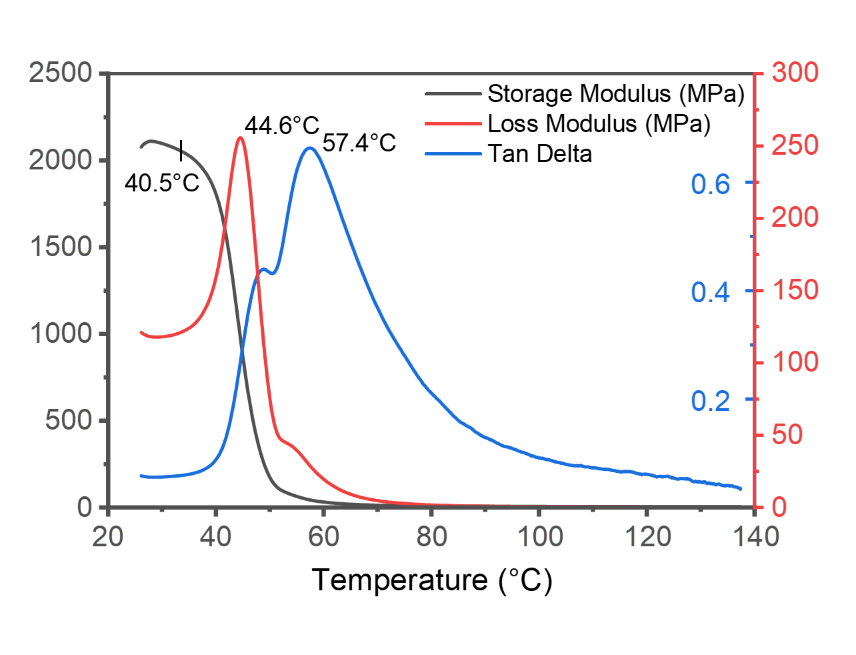


Figure S17. DMA analysis of unheated CAF-L5% film.


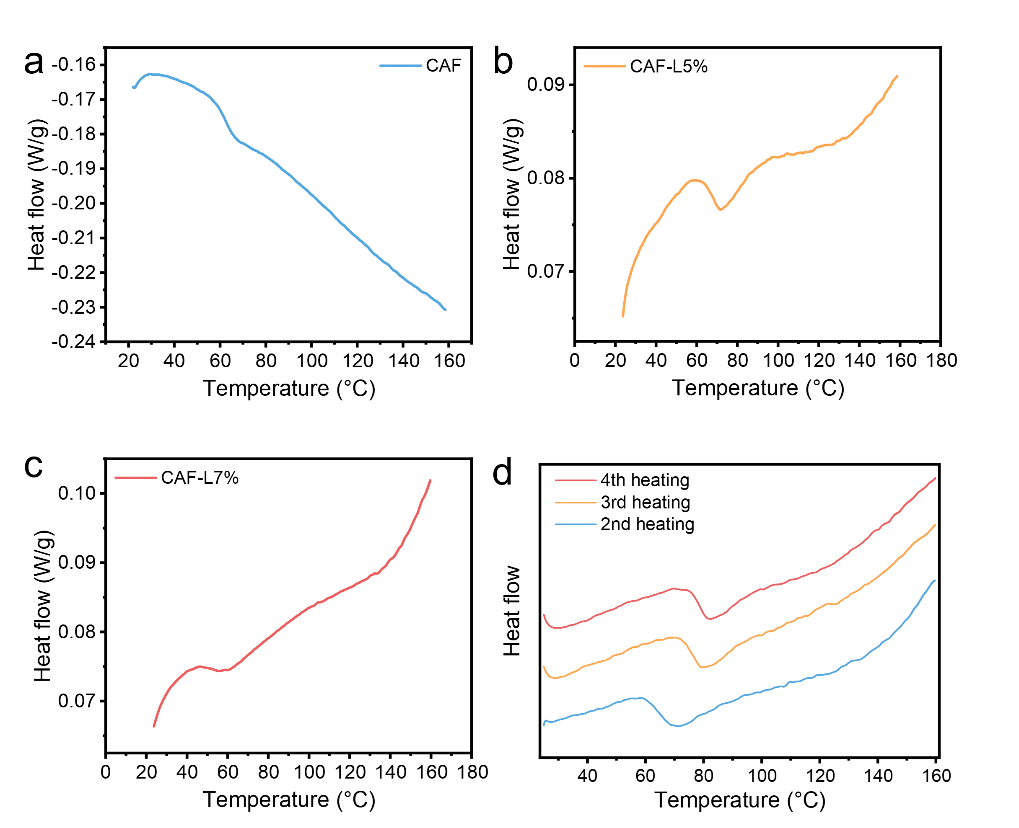


Figure S18. DSC curves of (a) CAF, (b) CAF-L5%, and (c) CAF-L7% films. (d) Multi-cycle DSC heating curves of CAF-L5% after removal of thermal history.


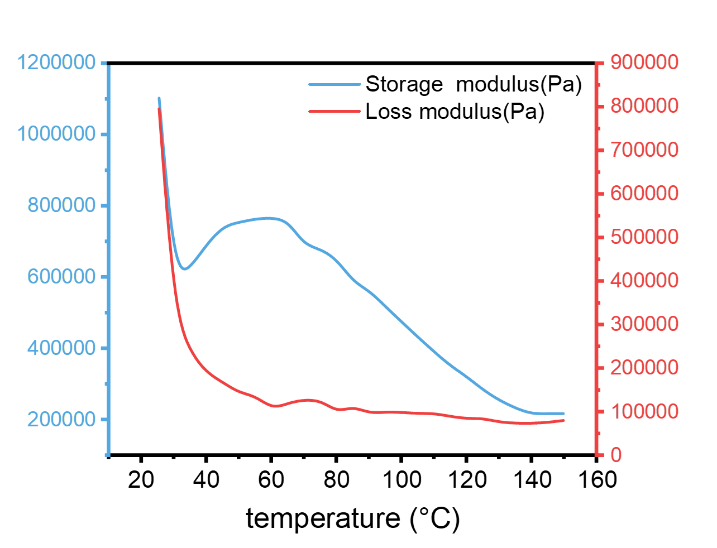


Figure S19. Temperature-sweep rotational rheology curves of CAF-L7% films under plate geometry.


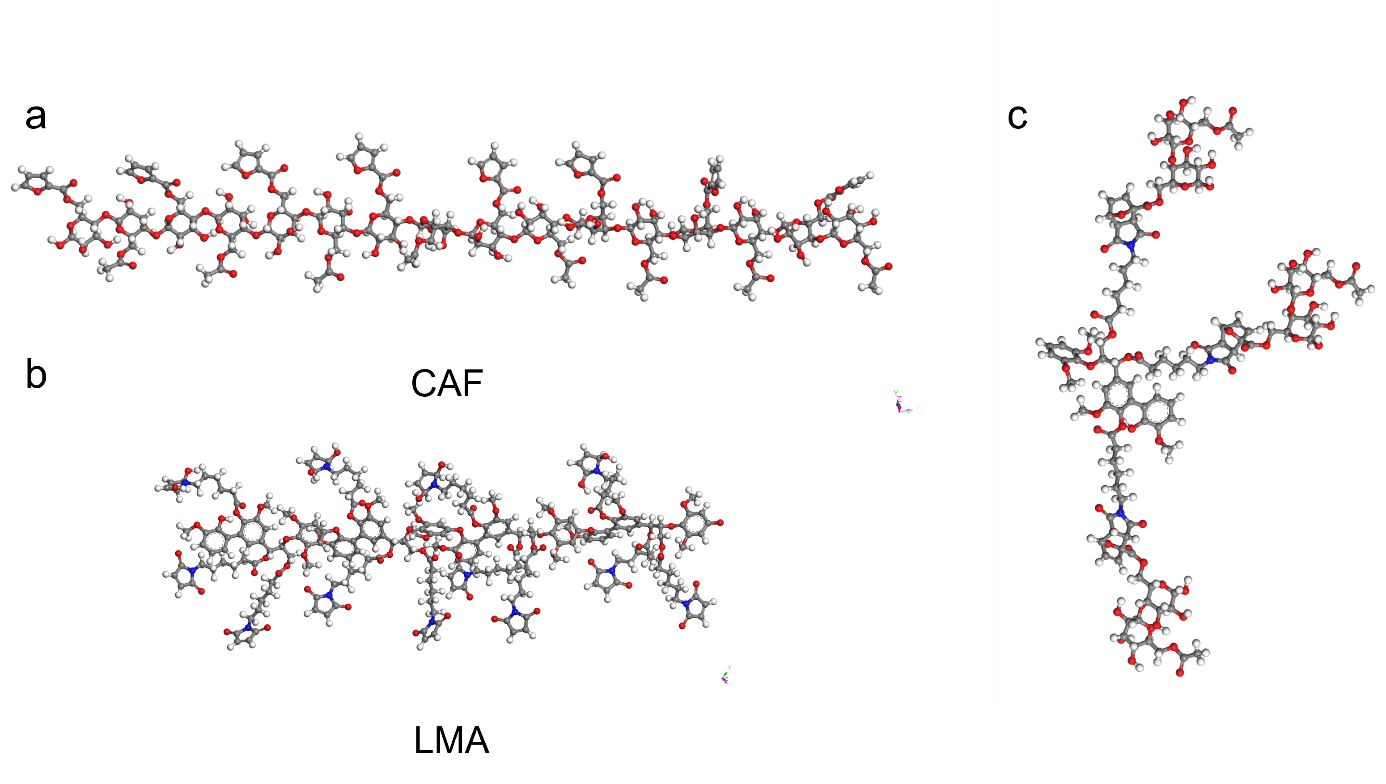


Figure S20. Molecular dynamics-optimized models of (a) CAF and (b) LMA. (c) Localized view of the CAF-LMA crosslinking model in molecular dynamics simulations.


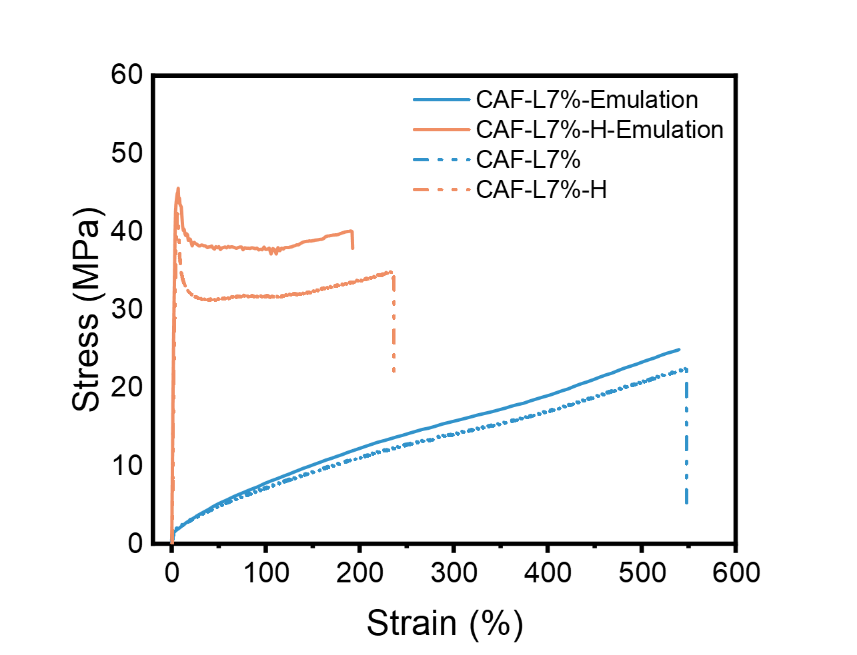


Figure S21. Finite element simulation and experimental stress–strain curves of CAF-L.


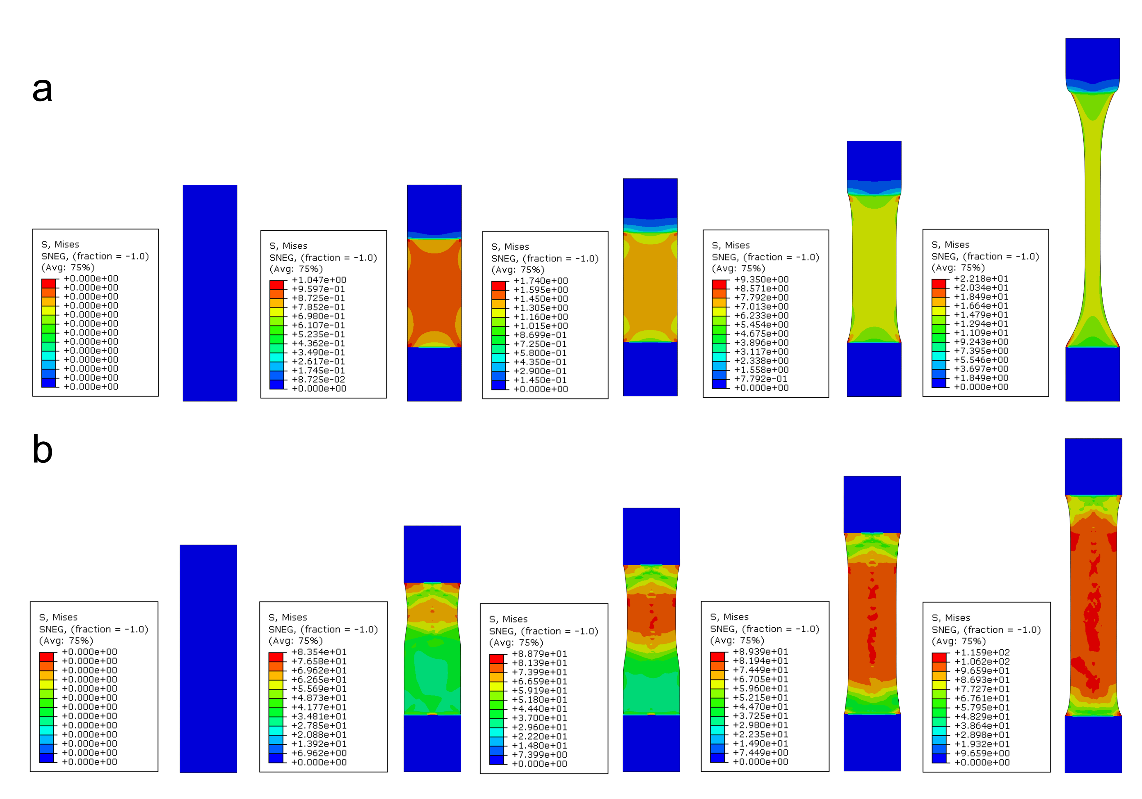


Figure S22. Finite element simulation of stress distribution during the tensile process of (a) CAF-L and (b) CAF-L-H films.


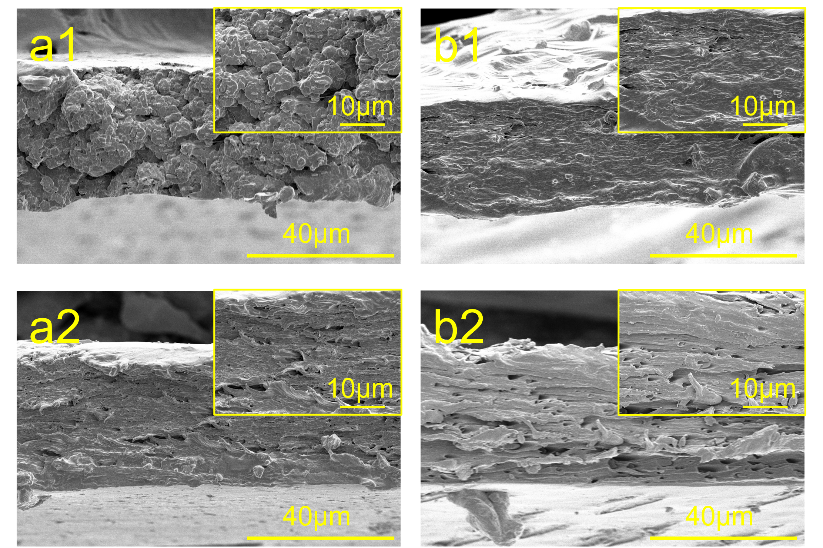


Figure S23. SEM images at various magnifications of cross-sections for (a) CAF-L3% and (b) CAF-L5%: (a1, b1) before heat-treatment and (a2, b2) after heat-treatment.


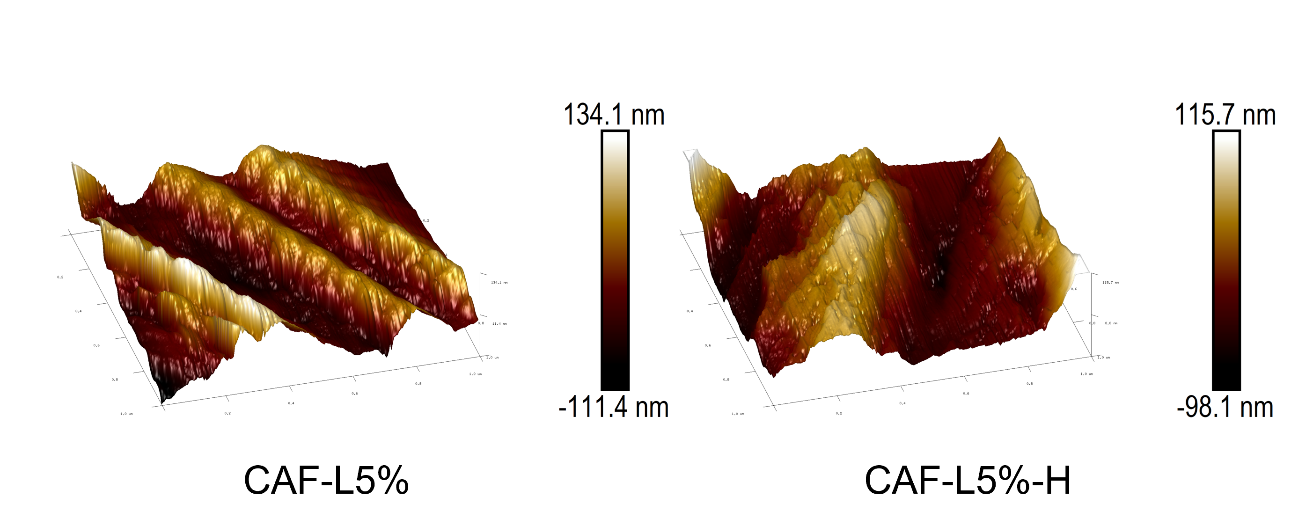


Figure S24. AFM height images of the tensile fracture surfaces of CAF-L7% films before and after heat treatment.


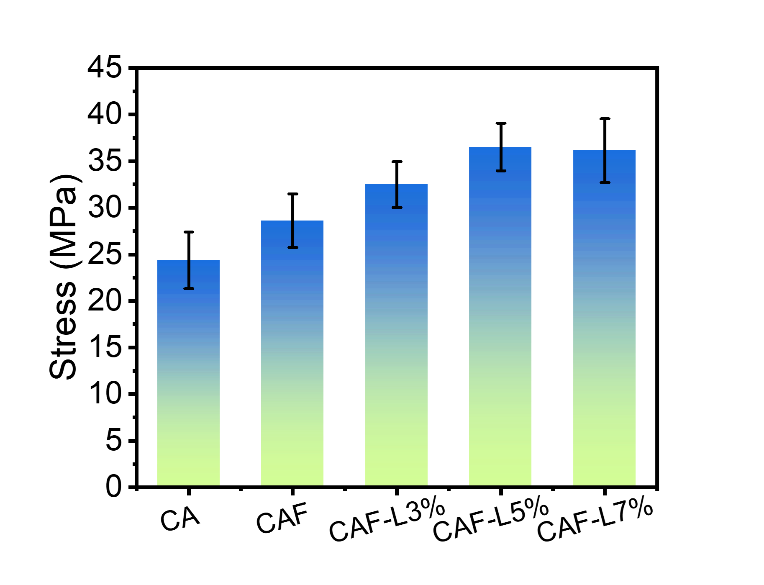


Figure S25. Heat seal strength data for CA, CAF, CAF-L3%, CAF-L5%, and CAF-L7% films.


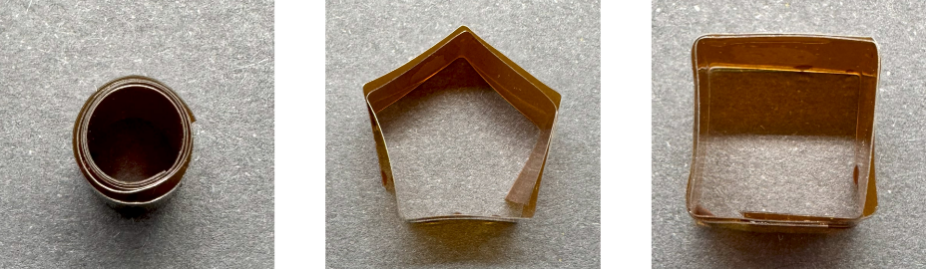


Figure S26. Digital photographs of CAF-L shape memory images: circular, pentagonal star, and rectangular shapes.


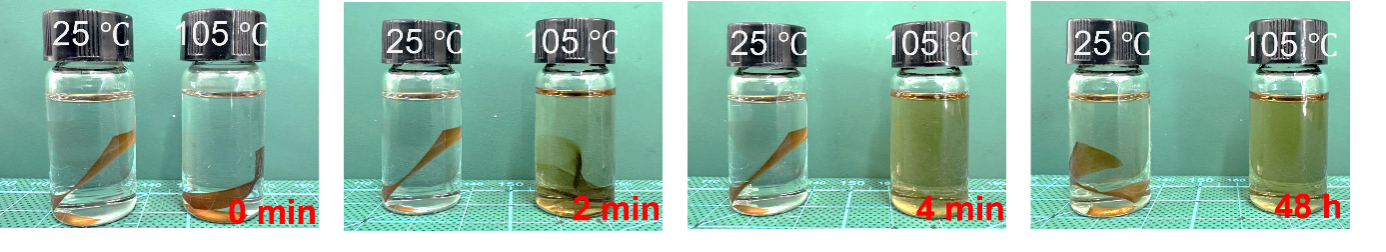


Figure S27. Recycling performance of CAF-L films in DMSO at room temperature and 105 °C.


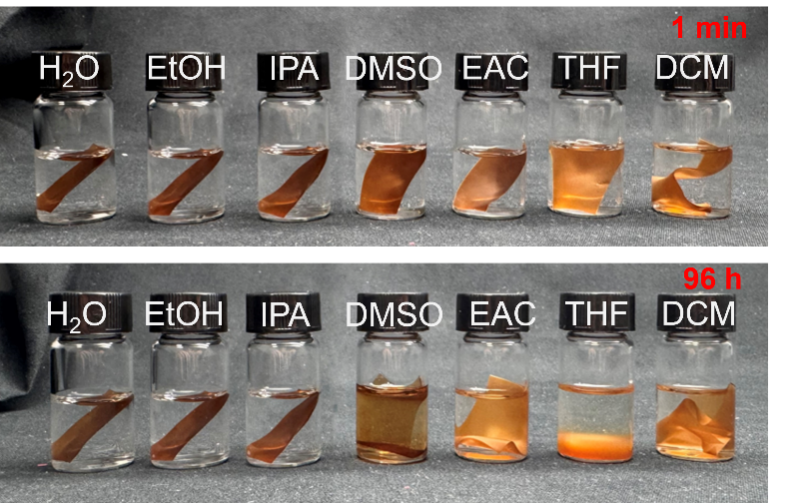


Figure S28. Digital photographs showing the stability of CAF-L in various solvents after 1 minute and 96 hours.
